# The intestinal microbiome is a co-determinant of the postprandial plasma glucose response

**DOI:** 10.1371/journal.pone.0238648

**Published:** 2020-09-18

**Authors:** Nadja B. Søndertoft, Josef K. Vogt, Manimozhiyan Arumugam, Mette Kristensen, Rikke J. Gøbel, Yong Fan, Liwei Lyu, Martin I. Bahl, Carsten Eriksen, Lars Ängquist, Hanne Frøkiær, Tue H. Hansen, Susanne Brix, H. Bjørn Nielsen, Torben Hansen, Henrik Vestergaard, Ramneek Gupta, Tine R. Licht, Lotte Lauritzen, Oluf Pedersen

**Affiliations:** 1 Novo Nordisk Foundation Center for Basic Metabolic Research, University of Copenhagen, Copenhagen, Denmark; 2 Department of Nutrition, Exercise and Sports, University of Copenhagen, Copenhagen, Denmark; 3 National Food Institute, Technical University of Denmark, Lyngby, Denmark; 4 Department of Biotechnology and Biomedicine, Technical University of Denmark, Kgs. Lyngby, Denmark; 5 Department of Veterinary Disease Biology, Faculty of Science, University of Copenhagen, Frederiksberg, Denmark; 6 Clinical-Microbiomics A/S, Copenhagen, Denmark; 7 Department of Health Technology, Technical University of Denmark, Lyngby, Denmark; Wageningen Universiteit, NETHERLANDS

## Abstract

Elevated postprandial plasma glucose is a risk factor for development of type 2 diabetes and cardiovascular disease. We hypothesized that the inter-individual postprandial plasma glucose response varies partly depending on the intestinal microbiome composition and function. We analyzed data from Danish adults (n = 106), who were self-reported healthy and attended the baseline visit of two previously reported randomized controlled cross-over trials within the Gut, Grain and Greens project. Plasma glucose concentrations at five time points were measured before and during three hours after a standardized breakfast. Based on these data, we devised machine learning algorithms integrating bio-clinical, as well as shotgun-sequencing-derived taxa and functional potentials of the intestinal microbiome to predict individual postprandial glucose excursions. In this *post hoc* study, we found microbial and clinical features, which predicted up to 48% of the inter-individual variance of postprandial plasma glucose responses (Pearson correlation coefficient of measured vs. predicted values, R = 0.69, 95% CI: 0.45 to 0.84, *p*<0.001). The features were age, fasting serum triglycerides, systolic blood pressure, BMI, fasting total serum cholesterol, abundance of *Bifidobacterium* genus, richness of metagenomics species and abundance of a metagenomic species annotated to *Clostridiales* at order level. A model based only on microbial features predicted up to 14% of the variance in postprandial plasma glucose excursions (R = 0.37, 95% CI: 0.02 to 0.64, *p* = 0.04). Adding fasting glycaemic measures to the model including microbial and bio-clinical features increased the predictive power to R = 0.78 (95% CI: 0.59 to 0.89, *p*<0.001), explaining more than 60% of the inter-individual variance of postprandial plasma glucose concentrations. The outcome of the study points to a potential role of the taxa and functional potentials of the intestinal microbiome. If validated in larger studies our findings may be included in future algorithms attempting to develop personalized nutrition, especially for prediction of individual blood glucose excursions in dys-glycaemic individuals.

## Introduction

More than 451 million people suffer from diabetes worldwide and the number is expected to increase to 693 million by 2045 [1]. Postprandial hyperglycaemia is a risk factor for the development of type 2 diabetes and cardiovascular disease [2]. Recently, there has been considerable interest in the inter- and intra-individual differences in postprandial plasma glucose responses (PPGRs) to gain insight into the mechanisms that underlie the dynamic responses and apply this information to tailor individualized recommendation on lifestyle and potential pharmaceutical intervention [3, 4]. The massive increase in data flow combined with accelerating computational capacity and bioinformatics expertise have paved the way for uncovering patterns not previously recognized with conventional statistical approaches.

A landmark study in the field involved about 800 healthy and pre-diabetic Israeli individuals, who consumed identical standardized meals [3]. The investigators demonstrated that PPGRs vary considerably among and within study participants, and constructed an algorithm that accurately predicted PPGRs to a variety of meals with the gut microbiota composition and functional potential contributing to the model [3]. Testing the algorithm in a US population showed that it was applicable to this population as well. The developed computational frameworks outperformed models considering solely the calorie or carbohydrate content of the meals consumed; traditional approaches, which are commonly used for prediction of blood glucose responses [4]. However, only relatively few details on the microbiome contributions to the predictive value of the models were reported.

During the recent decade, aberrant gut microbiomes have gained increased attention showing direct correlations with inflammatory and metabolic diseases such as obesity [5, 6] and type 2 diabetes [7–9]. Similarly, potential mechanistic links between altered gut bacteria and metabolic dysfunctions have been suggested from faeces inoculation experiments [10–12]. Still, a substantially deeper understanding of the dynamics and interplay of the gut microbiome and host metabolism requires deciphering of the specific intestinal bacterial taxa or functional potentials that drive the link to PPGRs and the magnitude of the effect of the gut microbiota on PPGRs. Particularly, non-linear methods and deliberate feature reduction methods are essential when analyzing a complex community such as the gut microbiome characterized by zero-inflation, overdispersion, heterogeneity and high-dimensionality.

**Fig 1:**
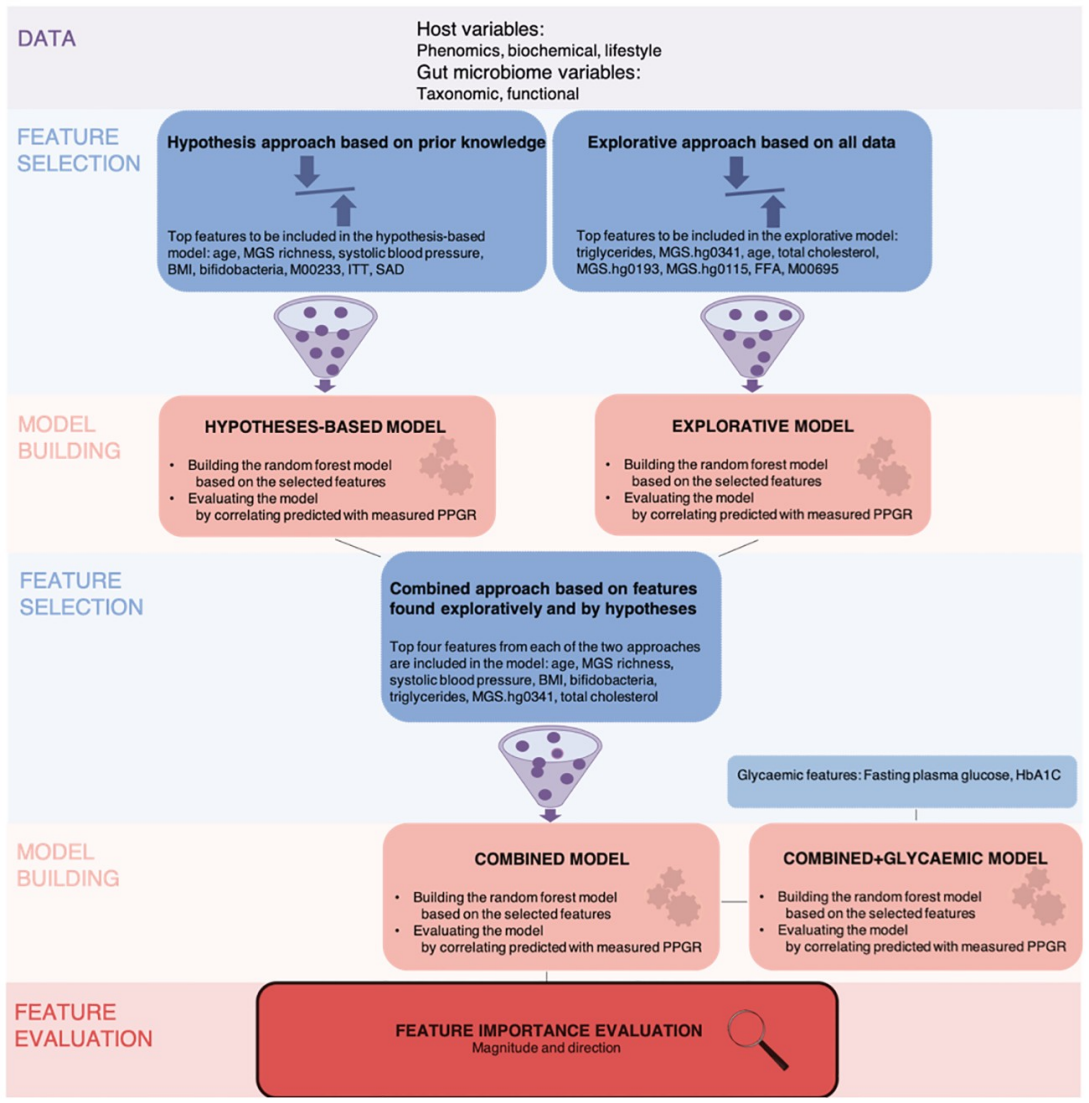
Data overview and experimental design. Host variables including phenomics, biochemical and lifestyle and gut microbiome variables were summarized as functional and species abundance profiles adjusted for bacterial cell counts of faecal samples. Data were randomly split into training (70%) and test (30%) sets. By two approaches, a hypothesis-driven and an explorative approach, features were selected and ranked according to the number of times selected in 200 rounds of top-down searches for important features applying wrapper algorithms build around random forest models predicting postprandial plasma glucose responses. In the hypothesis-based approach the number of features were reduced from 36 features selected from *a priori* knowledge to eight features, whereas in the explorative approach eight features were selected from all of the data comprising a total of 1368 features. For each approach, a random forest model predicting postprandial plasma glucose responses based on the eight selected features was trained and evaluated on the test set by correlating predicted with measured postprandial plasma glucose excursions. A combined random forest model predicting postprandial plasma glucose responses was trained based on the top features from each feature selection results thereby exploiting both approaches. The combined model was evaluated on the test set by correlating predicted with measured postprandial plasma glucose responses and the importance of the predictor features in terms of magnitudes and directions were evaluated individually and for the microbiome features combined. Finally, glycaemic features were added to the combined model to enable comparisons to previous findings in the literature.

We hypothesize that the inter-individual PPGRs vary partly depending on the intestinal microbiome composition and function. In the present *post hoc* study in self-reported healthy Danish adults we investigated the relationship between the gut microbiome, clinical and lifestyle variables on one side, and plasma glucose excursions during three hours following a standardized breakfast on the other [13, 14]. We applied a machine learning approach and report the drivers of the PPGRs with focus on the contributions from the gut microbiome. Our random forest algorithm included phenomics, biochemical, lifestyle and gut microbial features selected based on prior knowledge and by an explorative search to improve model optimization for a cohort with limited size (Fig 1 and S1 Table). We evaluated the importance of the models in terms of magnitude and direction of the individual features. For comparison, we evaluated a model including glycaemic features in the fasting state.

## Materials and methods

### Study design

The present study was based on analyses of baseline data from the Gut, Grain and Greens (3G) project comprising two randomized controlled cross-over trials conducted in Denmark. Detailed descriptions of the objective, design and methods of the clinical trials have been reported [13, 14].

Importantly, participants had to meet similar inclusion and exclusion criteria in both trials and the different modes of interventions in the two studies are irrelevant for the present study, which exclusively was based on analyses of data obtained at baseline prior to start of the specific interventions [15]. The trials were registered at www.clinicaltrials.gov (NCT01719913 and NCT01731366) and were conducted in accordance with the Helsinki declaration II and endorsed by the Danish Data Protection Agency (2007-54-0269) and The Ethical Committee of the Capital Region of Denmark approved the trials (H-2-2012-065). All individuals gave written informed consent. The trials were conducted from July 2012 to November 2013. Only participants who provided a faecal sample in relation to baseline examination were included in the present study.

### Phenomics, biochemistry and lifestyle variables

The collection of bio-clinical features including phenomics, biochemical variables and information on dietary and smoking habits has been reported [13, 14]. Body weight, height, sagittal abdominal diameter and blood pressure (after 15 min rest) were measured. Body composition was measured by bioelectrical impedance analysis (QuadScan 4000, Bodystat Inc., Isle of Man, UK) and fat-free mass was calculated by subtracting fat mass from body weight. Intestinal transit time was assessed by abdominal X-ray after participants ingested nonabsorbable radio-opaque transit markers, and calculated based on the number of visible markers on the obtained abdominal X radiographs, adjusted for time since last marker ingestion [16, 17]. Dietary intake of macronutrients was assessed from a validated four-day pre-coded dietary record [18]. Stool consistency was self-assessed according to a seven-point Bristol stool scale [19]. Smoking habits were categorized as current smoker or non-smoker.

### Meal test and biochemical variables

The methods related to the standardized meal test were previously reported [13, 14]. The participants were given a standardized breakfast, which consisted of white wheat bread, a pastry, butter, jam, cheese and 200 mL water (~3000 kJ, 52 E% fat, 40 E% carbohydrate, 8 E% protein, 3 g dietary fiber per 100 g) and a drink containing lactulose (5 g) and mannitol (2 g). Blood samples were drawn after a rest of at least 10 min in the fasting state in the morning (t = 0 min) and postprandially (t = 30, 60, 120 and 180 min) after consumption of the meal. Plasma glucose, HbA1c and serum triglycerides, total-, low-density lipoprotein-, and high-density lipoprotein -cholesterol, plasma free fatty acids, alanine aminotransferase and aspartate aminotransferase were analyzed using automated, enzymatic, colorimetric assay on ABX Pentra 400 chemistry analyzer (ABX Pentra, Horiba ABX, Montpellier, France). The response variable of our analyses, PPGR, was calculated as the total area under the curve using the composite trapezoid rule applying the R package “MESS” [20]. Serum insulin and C-peptide were measured by a chemiluminescent immunometric assay (Immulite 1000; Siemens Medical Solutions Diagnostics, Los Angeles, USA). High-sensitivity serum C-reactive protein (hsCRP) was measured after a 1000× dilution in a high-sensitivity single-plex assay (MesoScale Discovery^®^, Gaithersburg, MD, USA) using the Sector Imager 2400A (MesoScale Discovery^®^). Serum interleukin-6 and tumor necrosis factor-α were measured by high-sensitivity enzyme-linked immunosorbent assays (ELISA) (R&D systems, Minneapolis, Minnesota, USA, HSLB00C, HS600B, and HSTA00D). Serum zonulin was measured using IDK Zonulin ELISA kit (Immundiagnostik AG, Bensheim, Germany). Intact glucagon-like peptide-2 was measured using an in-house developed radioimmunoassay [13].

### Gut microbiome data

Faecal sample collection, bacterial DNA extraction and shotgun-based metagenomics sequencing were previously reported [13, 14]. In brief, the faecal samples were collected in the morning and immediately stored at 5°C for a maximum of 24 h before equal volume of sterile water was added and after thawing, the sample was homogenized. The homogenized sample was aliquoted to cryotubes, and stored at −80°C. Microbial DNA was extracted from faeces [21] and metagenomic sequencing was performed on HiSeq 2000 (Illumina Int., San Diego, CA USA) at BGI Tech Europe (BGI Tech Europe, Copenhagen, Denmark) with a median of 70177376 (IQR: 19443411) readouts per sample (100 bp paired-end) (detailed information in [13, 14]). We included data on quantitative microbiome profiles (QMP), which were generated after the publication of the two core papers on the clinical trials [13, 14]. Details of the procedures for QMP are described in (S1 Appendix). Briefly, after having been thawed, the frozen faecal samples were divided into aliquots and diluted in staining buffer. Debris was removed from the faecal solutions and a fixed amount of the microbial cell suspension was stained for flow cytometry analysis. Fluorescence events were monitored and forward- and sideways-scattered lights were collected. The microbial fluorescence events were gated and seperated from the faecal sample background. The resulting counting numbers were used to rarefy to equal sampling depth for quantitative analyses of microbiome features [22]. The detailed bioinformatic characterization of the gut microbiota is described in (S1 Appendix). Briefly, quality control was done to remove low-quality bases and reads derived from the host genome. The reads were mapped to an extensive gene catalogue (20 041 521 genes, >13 735 303 172 bp) constructed of multiple publicly available microbiome studies (S1 Appendix). The method for creating the catalogue was described previously [23]. The metagenomic species (MGS) assignment was adapted from Nielsen *et al*. [24]. For each MGS, the signature genes were defined as 100 genes optimized for accurate abundance profiling of the MGS. An MGS counts table was created based on the count of reads mapped to each of the MGS signature gene sets. This MGS counts table was normalized according to effective gene length (accounting for read length) and subsequently rarefied according to QMP. An MGS present in less than 10 individuals was excluded from the analyses. Samples were downsized to an adjusted sampling depth, defined as the ratio between sample size and microbial load (average total cell count/g of frozen faecal material). Thus, the sequencing depth of each sample was rarefied to the level necessary to equalize the minimum observed sampling depth in the cohort (median ratio: 168498 [119264; 214955]). The tools and procedure are available from Professor Jeroen Raes’ Lab, Belgium (http://www.raeslab.org/software/QMP) [22] with an R script at https://github.com/raeslab/QMP/blob/master/QMP.R using the phyloseq R package [25]. MGS richness and diversity were calculated by means of Shannon index and richness as number of MGS detected per rarefied sample. Microbial gene richness was calculated as the number of microbial genes detected per sample. For functional annotation, the genes were annotated to the EggNOG database (v. 4.5) and the KEGG database (http://www.genome.jp/kegg/kegg1.html) using the MOCAT2 lookup tables (http://mocat.embl.de/). The KEGG Orthologies (KOs) were then rarefied according to QMP. The rarefied KOs were grouped into KEGG modules [26] based on 66% presence of the KOs in the module. The KEGG modules were then normalized according to module length. The KEGG modules were excluded from the analyses if present in less than 10 individuals. The rarefied KOs were also grouped into manually curated modules (Gut Metabolic Modules) with a minimum coverage of 0.66 [27]. The modules are available from Raes Lab, Belgium (http://www.raeslab.org/software/gmms.html) and generation of these (https://github.com/omixer/omixer-rpmR/blob/master/README.md) was done by use of the R package omixerRpm [28].

### Statistical analyses

Before analyses, data were randomly split into training (70%) and test (30%) sets. Feature selection was done by applying an all-relevant feature selection wrapper algorithm (R package Boruta) 200 times in the training set to restrict the complexity of the learned model due to the small size of the study population. Boruta may be particularly attractive in biomedical applications, where often the primary interest is to determine all of the features, which are relevant for a biological condition such as PPGRs. Specifically, Boruta estimated feature importance achievable at random by using features’ permuted copies and compare with the importance of original features in a number of random forest models. Features were then progressively marked as relevant or irrelevant [29]. Random forest is an ensemble model consisting of multiple trees built in many bootstrap samples that offers opportunities to find non-linear patterns in heterogeneous and high-dimensional datasets with few samples [30]. The prediction accuracy of random forest as applied to microbiome-host trait prediction was evaluated as either the most accurate or competitive when compared with the most commonly used machine learning methods [31]. The optimal number of features in a random forest model may be context-specific leaving no universal answer. However, simulation studies for classification models suggest the square-root of the training population [32]. Thus, models included eight features when possible to improve model optimization with a limited study sample size. Building and tuning the random forest regression models were done in the open source machine learning platform H2O [33]. We tuned the model in the training set using a hyperparameter grid (S1 Appendix) and included five-fold cross-validation to minimize overfitting and improve generalizability. For each model, the best parameter set was determined by the lowest expected prediction error, i.e. mean squared error. The grid search continued until none of the last 10 models showed a 0.5% improvement in mean squared error compared with the best previous model or after a running time of 120 min. Performance of all models were assessed by correlating the predicted values and the measured values by Pearson correlation using the R package ggpubr [34]. Furthermore, to test for overfitting, we did a new feature selection based on the randomly shuffled prediction outcome and trained a model based on the new features found. Training a model on permuted responses reveal whether the new model performs better than random. The contributions from individual features in the model were evaluated in terms of ranking according to importance and directionality of the effect. Feature importance is by default determined by calculating the relative effect of each feature based on whether that feature was selected to split on, and how much the mean squared error changed as a result [33]. To demonstrate the certainty of this feature importance, 700 models with identical hyperparameters, but different random model training seed, were trained and confidence intervals for each predictor feature importance were reported. However, this default method is less accurate and not as reliable in situations where potential predictor features vary in scale of measurement or their number of categories compared to other methods, as it has a tendency to inflate the importance of continuous or high-cardinality features [35]. Another approach is to use the cross-validation or bootstrap samples to estimate the variability in randomly labeled data, and evaluate the prediction accuracy. Specifically, we have trained a model with identical hyperparameters as the combined model but with the feature of interest being permuted. The absolute values of the residuals, i.e. the pure positive deviations from the known ‘true’ values for both models of each feature, respectively, were calculated and these values were compared in a paired t-test. In addition, ranking was done by “drop one column”, i.e., one feature was removed from the model and the model was retrained and evaluated. Subsequently, the importance of a feature was calculated as the difference between the model performance with or without the feature. This provides a more accurate and less biased feature importance even though correlated features may appear less important since the effect is maintained in the model. This feature importance was plotted by use of the R package ggplot2 [36]. The directionality of the effect was evaluated by partial dependence plots applying the R package H2O and ggpubr [33, 34]. Partial dependence plots are graphical depictions of the marginal effect of a feature on the response outcome when the average effects of the other features are accounted for [37]. While the plots may suggest feature contributions, it may also be misleading due to higher-order correlations [37]. No multiple test correction was done due to the use of both training and test sets and the low number of statistical tests. Values >10 mg/L for high-sensitivity serum C-reactive protein concentrations were removed (n = 4). Missing data was handled by median imputation (~0.08% of all values). All statistical analyses were performed in R version 3.5.2 [38].

## Results

### Baseline characteristics of study participants

The study population comprised 106 Danish adults, who were healthy by self-report, attended the baseline visit and provided a faecal sample (56 from the low-gluten trial and 50 from the whole grain-rich trial of the Gut, Grain and Greens project (3G) study cohort [13, 14]). The study sample was randomly split into a training and a test set including 75 and 31 participants, respectively (Table 1).

**Table 1:** Baseline characteristics of study participants in the training and test sets.

	Training set	Test set
**n**	75	31
**Age (years)**	50.5 (11)	45.1 (12)
**Female, n (%)**	45 (60)	19 (63)
**BMI (kg/m**^**2**^**)**	29 (3)	29 (3)
**Fasting serum triglycerides (mmol/l)**	1.1 [0.9, 1.7]	1.1 [0.9, 1.6]
**Fasting serum HDL cholesterol (mmol/l)**	1.3 (0.3)	1.4 (0.3)
**Systolic BP (mmHg)**	127 (13)	127 (12)
**Fasting plasma glucose (mmol/l)**	5.7 (0.6)	5.6 (0.6)
**HbA1c (mmol/mol)**	36.3 (2.5)	35.4 (2.7)
**HbA1c (%)**	3.3 (0.2)	3.2 (0.3)
**Area under the curve (AUC) of plasma glucose (mmol×3h/l)**	1126 (172)	1132 (164)

Summary statistics of physiological traits. Data are presented as means (SD) or median [IQR] in case of non-normal distributions.

The mean PPGR, calculated as area under the response curve to the standardized breakfast, was 1128±169 mmol/l in the study population and varied substantially between individuals with peak concentrations ranging from 4.7 to 11.2 mmol/l (S1 Fig). The overall analytical workflow is depicted in Fig 1. In short, features either from a subset of features with *a priori* hypotheses (hypothesis-based approach: 36 features) or the entire dataset (explorative approach: 1368 features) were selected and ranked according to importance relative to PPGRs. Random forest models predicting PPGRs were trained and evaluated for both approaches; followed by a combined model trained based on the top four features from each feature selection approach. The importance of the features selected for the combined prediction model in terms of magnitude and direction were evaluated individually and for the microbiome features combined. Finally, glycaemic features collected during the fasting state were added to the combined model to enable comparisons to previous findings in the literature.

### Hypothesis-driven analytical approach

#### Selection of features to be included in the hypothesis-driven analysis of determinants of postprandial plasma glucose responses

In the hypothesis-driven analyses of variables related to PPGRs, a list of 36 features relevant for PPGRs was created based on reported prior knowledge (S2 Table). Nine features were confirmed as important in 200 feature selection rounds and were ranked according to number of times of appearance in the rounds [29]. The eight features included in the hypothesis model were age, MGS richness, systolic blood pressure, BMI, abundance of the *Bifidobacterium* genus, abundance of KEGG module glutamate transport system (M00233), intestinal transit time and sagittal abdominal diameter (S3 Table, S2 Fig).

A random forest-based algorithm was built using five-fold cross-validation based on *a priori* selected features (S4 Table). Pearson coefficient of the correlation between predicted and measured PPGRs was R = 0.42 for the test set of 31 samples (*p* = 0.02, 95% CI: 0.07 to 0.67) (S3 Fig).

### Explorative analytical approach

#### Selection of features to be included in the explorative analyses of determinants of postprandial plasma glucose responses

In parallel, from the 1368 features including phenomics, biochemical variables, gut microbiome data and information on lifestyle, the top eight hits were selected to enter the explorative model based on the results from 200 feature selection rounds. The selected features were fasting serum triglyceride concentration, the abundance of three MGS’s annotated at the order level as *Clostridiales* (MGS.hg0341, MGS.hg0193 and MGS.hg0115), fasting total serum cholesterol concentration, age, plasma free fatty acids and abundance of KEGG module cAMP signaling (M00695) (S5 Table, S4 Fig).

In the predictive model analysis, a random forest algorithm was built based on the selected features (S6 Table). Pearson coefficient of the correlation between predicted and measured PPGRs was R = 0.66 for the test set (*p*<0.001, 95% CI: 0.40 to 0.82, S5 Fig).

### Combined analytical approach

#### Selection of features for inclusion in a combined random forest-based model analysis

**Fig 2:**
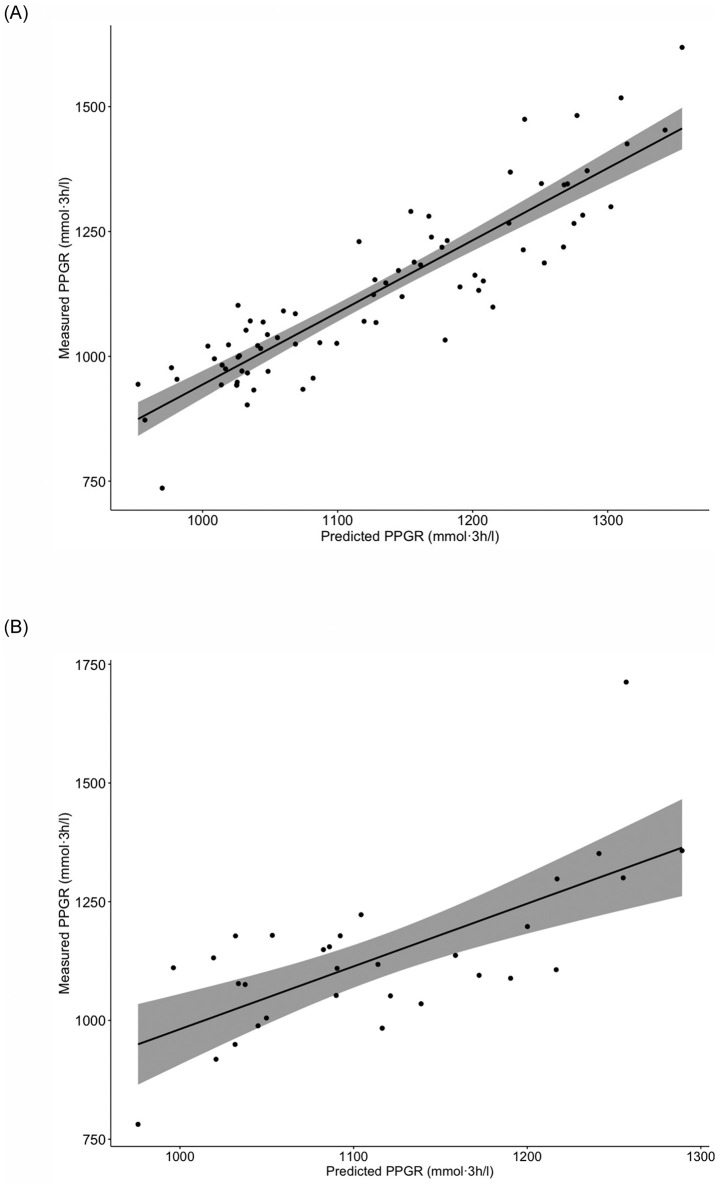
Association between predicted and measured postprandial plasma glucose. Predictions are based on the combined model including the following features: fasting serum concentration of triglycerides, age, fasting total serum cholesterol concentration, abundance of MGS.hg0341, abundance of bifidobacteria, BMI, systolic blood pressure and MGS richness. The black line represents the fitted regression line and the grey shaded area represents the 95% CIs. (A) The association in the training set (n = 75). Pearson R = 0.90, 95% CI: 0.84 to 0.93 and *p*<0.001. (B) The association in the test set (n = 31). Pearson R = 0.69, 95% CI: 0.45 to 0.84 and *p*<0.001.

In an attempt to construct an optimized model based on a combination of the features identified in the hypothesis-driven and the explorative approach, respectively, we selected the top ranked features from each of the two feature selection outcomes. Since age was part of the selected features by both approaches, the top five features of the hypothesis-driven approach were selected in order to have eight features for the combined model. The eight features were age, MGS richness, systolic blood pressure, BMI, abundance of the *Bifidobacterium* genus, fasting serum triglyceride concentration, the abundance of one MGS annotated at the order level as *Clostridiales* (MGS.hg0341) and fasting serum total cholesterol concentration. The combined model containing the outlined features outperformed the hypothesis-driven and the explorative models (S7 Table) with a Pearson correlation between predicted and measured PPGRs of R = 0.69 for the test set (*p*<0.001, 95% CI: 0.45 to 0.84) (Fig 2).

#### Feature contributions

**Fig 3:**
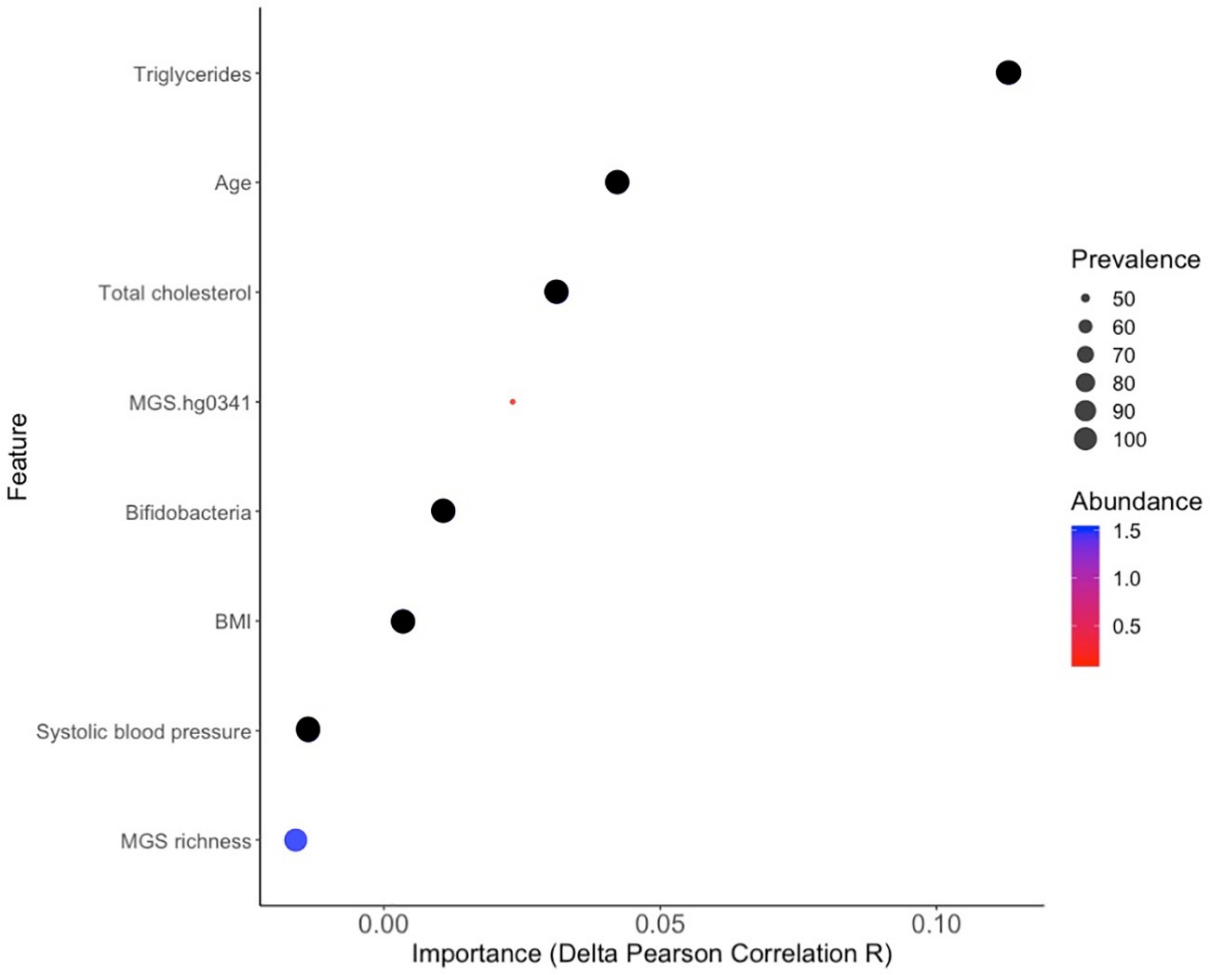
Contributions of all features in the combined model. Contributions are calculated by removing the feature of interest from the combined model, retrain, evaluate the model by correlating measured and predicted postprandial plasma glucose responses in the test set and subsequently subtract the Pearson correlation R from the one obtained from the combined model including all of the features (delta R). Circles are scaled according to prevalence (prevalence refers to the number of individuals harboring the microbiome feature in %) and coloured according to abundance (abundance is the relative representation of the individual microbiome feature in the gut microbiome in %) (n = 31).

The contributions from individual features in the combined model were evaluated by ranking according to impact in the test set. Six of the features contributed to a stronger correlation between predicted and measured PPGRs compared with a model missing this feature. The features age and fasting serum triglyceride concentration had the highest impact (S8 Table, Fig 3), whereas systolic blood pressure and MGS richness did not contribute to a more accurate prediction. Applying the drop-one column approach in the test set has the advantage of showing a more generalizable performance information being more similar to ‘real life’ performance.

The feature importance was reported in the training set by default as well, together with the hyperparameters in the model (S7 Table). The confidence intervals for these feature importances of age and MGS.hg0341 overlapped reflecting that there is some uncertainty of second or third rank. Moreover, we found that none of the features contributed significantly to the predictions when comparing the absolute value of the residuals (predictions subtracted from true values) from the combined model and the combined model where the feature of interest was permuted. This may indicate that no feature is by its own driving the model predictions and that the features in the model correlate.

Overall, the different approaches for reporting feature contributions yielded similar results overall in terms of ranking of importance of features in the training and test sets.

**Fig 4:**
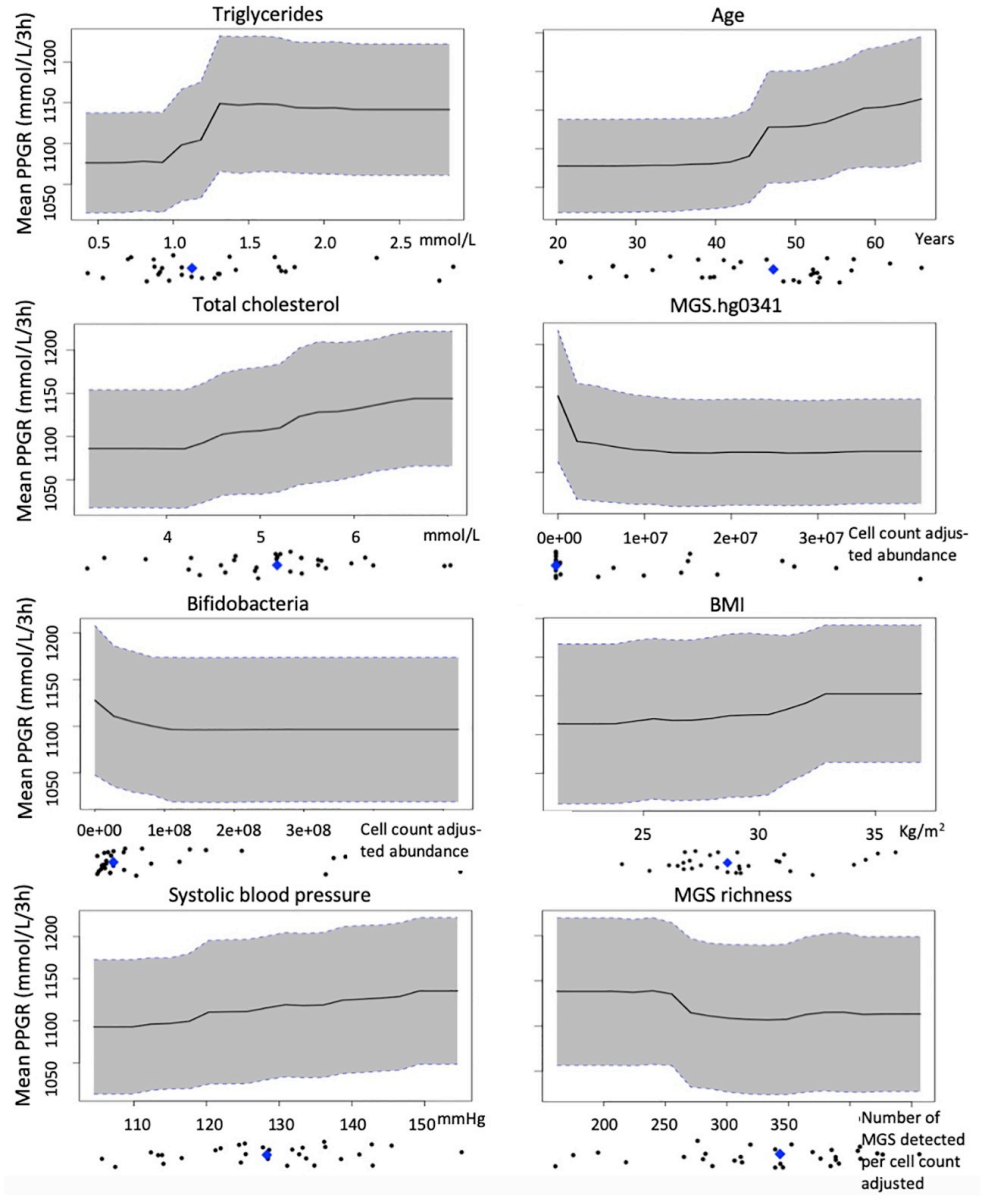
Partial dependence plots showing the marginal contribution of each feature in the combined model to the predicted postprandial plasma glucose responses. A positive trend indicates a non-beneficial effect of the feature whereas a negative trend indicates a beneficial effect. Dot plots indicate the distributions of each feature in the study population, with the median is indicated by a blue diamond (n = 31).

The directionality of the effect was evaluated by partial dependence plots, which show the marginal effect of one feature on the predicted outcome of the model including the distribution of the feature in the study population. From the plots, it was evident that increasing values of fasting serum concentration of triglycerides, age, fasting total serum cholesterol concentration, systolic blood pressure and BMI were linked to increases of PPGRs, whereas increasing values of abundance of *Bifidobacterium* genus and MGS.hg0341 were linked to declines in PPGRs. MGS richness appeared to have a bimodal association to PPGRs (Fig 4).

We also plotted the correlation between each feature and the outcome individually to disclose the association including the strength and the association of each feature and PPGRs (S6 Fig).

#### Model robustness

To test the robustness of the presented optimized approaches (Boruta feature selection and random forest modelling), we next performed a permutation of the PPGR values. The top eight features generated from the Boruta feature selection in the training set were modelled and evaluated in the test set (S9 Table, S7 Fig). This random analysis gave a Pearson coefficient of the correlation between predicted and measured PPGRs of R = -0.13 (*p* = 0.51, 95% CI: -0.47 to 0.25, S10 Table, S8 Fig). Hence, this model was unable to predict PPGRs, which was in contrast to the results based on the combined model (*p* = 0.51 vs. *p*<0.01).

#### The intestinal microbiome is a part of the determinants of postprandial plasma glucose responses

To learn about the importance of the three above listed microbial features in the combined model, we excluded the phenomics, biochemical and lifestyle features from the model and evaluated a microbiome-only model (S11 Table). Pearson correlation between predicted and measured PPGRs was R = 0.37 for the test set, corresponding to 14% of the variance in PPGRs (*p* = 0.04, 95% CI: 0.02 to 0.64, S9 Fig).

#### Phenomics, biochemical and lifestyle variables that impact the combined algorithm predicting postprandial plasma glucose responses

The same approach was applied to a bio-clinical features-only model, Pearson correlation between predicted and measured PPGRs was R = 0.69 for the test set, corresponding to up to 48% of the variance in PPGRs (*p*<0.001, 95% CI: 0.45 to 0.84, S12 Table).

#### Combined model analysis including glycaemic variables in the fasting state

To be able to compare our results to the outcome of comparable analyses reported in the literature in which glycaemic variables were integrated, we included fasting concentrations of HbA1c and fasting plasma glucose into the combined model [3, 4]. Doing so, we obtained a Pearson correlation between predicted and measured PPGRs of R = 0.78 for the test set corresponding to 61% of the variance in PPGRs (*p*<0.001, 95% CI: 0.47 to 0.85, S10 Fig, S13 Table).

**Fig 5:**
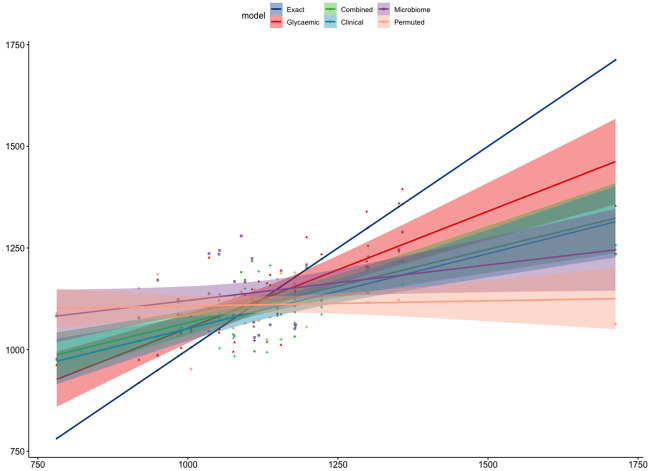
Comparison of the association strengths between the actual and predicted postprandial glucose responses in the test set based on the five different models and a perfect correlation as a comparison (exact); the combined model including glycaemic variables in the fasting state, the combined model, the bio-clinical features-only model, microbiome-only model and the null model of the permuted glucose responses. The lines represent the fitted regression lines and the corresponding shaded area represent the 95% CIs for each model, respectively. Exact: Pearson R = 1. The combined model including glycaemic variables in the fasting state: Pearson R = 0.78, 95% CI: 0.59–0.89 and *p*<0.001. The combined model: Pearson R = 0.69, 95% CI: 0.45–0.84 and *p*<0.001. The bio-clinical features-only model: Pearson R = 0.69, 95% CI: 0.45–0.84 and *p*<0.001. The microbiome-only model: Pearson R = 0.37, 95% CI: 0.02–0.64 and *p* = 0.04. The null model of the permuted glucose responses: Pearson R = -0.13, 95% CI: -0.47.0.25 and *p* = 0.51.

In summary, the predictions, based on the combined model including the glycaemic variables in the fasting state, when correlated to the actual values, outperformed the predictions based on all other models. The combined model and the bio-clinical features-only model performed similarly well. The predictions based on the microbiome-only model correlated significantly with the actual values as opposed to the null model of the permuted glucose responses (Fig 5 and S11 Fig in the test set and S12 Fig in the training set).

## Discussion

By integrating data of the intestinal microbiome, phenomics, biochemistry and lifestyle of middle-aged Danes we developed prediction models for PPGRs after intake of a standardized breakfast, where model features were either selected from hypotheses inspired by prior knowledge or derived from an explorative search. We combined the models and generated a combined model, which we evaluated in the test set of PPGR data and assessed the relative contributions from the included features individually and from microbial features combined and bio-clinical features combined, respectively. We confirmed that PPGRs after a standardized breakfast vary to a major extent among adult self-reported healthy Danish individuals and found that up to 48% of the variance was predictable from microbial and bio-clinical features increasing to more than 60% of variance explained when adding glycaemic variables measured in the fasting state. Intriguingly, 14% of the inter-individual variance of PPGRs can be explained by an algorithm exclusively based upon intestinal microbiome features.

In the present datasets, MGS richness, the abundance of *Bifidobacterium* genus and MGS.hg0341 were the highest performing microbiome features in the models. MGS richness, however, did not contribute to an improved model performance in the combined model of eight features. The lack of contribution of MGS richness in the combined model may be due to that the model is slightly overfitted and the features in the model are correlated rather than MGS richness is without influence on PPGRs in our dataset. Indeed, MGS richness contributed to an improved performance in the model of only microbiome features, where the predicted PPGRs correlated significantly with the measured PPGRs. In agreement with the inverse association of MGS richness with PPGRs in our prediction models, another study reported that classifying individuals based on bacterial gene richness revealed two groups differing in overall adiposity, insulin resistance, dyslipidemia and inflammation with high bacterial richness representing the lean phenotype [5]. However, from inspecting partial dependence plots (Fig 4), it appears as if the association between bacterial gene richness and PPGRs is bimodal with richness negatively correlated with PPGR until a certain count of MGS where after richness becomes positively correlated with PPGR. A possible explanation for this may be found in the association between richness and intestinal transit time, where increased richness due to decreased intestinal transit time is accompanied by a shift in colonic metabolism from carbohydrate fermentation to protein catabolism, which in turn has been linked to cardiovascular disease [39, 40]. Increased abundance of the bifidobacterium genus appeared as beneficial in our prediction models. Accordingly, the abundance of bifidobacteria is lower in individuals with type 2 diabetes than in healthy individuals [41], as well as in obese compared with healthy individuals [42], and elevated amounts of bifidobacteria in infancy associates with normal weight in later ages [43]. The mechanisms that underlie the association between increased bifidobacteria abundance and host metabolism remain unclear. One potential link is related to the hypolipidaemic effects of bifidobacteria observed in animal and human studies [44, 45]. Bifidobacteria possess genes encoding bile salt hydrolases, enzymes that catalyze the deconjugation of glycine- or taurine-linked bile salts [46] and have been suggested to decrease serum lipid levels by promoting bile acid biotransformation *in vivo* [42]. Furthermore, the anti-inflammatory properties of bifidobacteria are well-known [47] and in mice, administration of prebiotics increased abundance of bifidobacteria and improved glucose metabolism, low-grade inflammation and reduced risk of body weight gain [48].

We identified abundance of MGS.hg0341 as ranking in top of the feature selections and it was included in the combined model. Unfortunately, it was not possible to annotate it to a deeper resolution than the order level by comparing its gene sequences to known genomes (S1 Appendix). Identifying this particular strain is of potential interest since it in our models appears as a driver of the interplay between the gut microbiome and PPGRs.

In the present datasets, age, systolic blood pressure, BMI, total serum cholesterol and fasting serum triglycerides were the highest performing clinical features in the models. The clinical features have well-established positive associations to PPGRs [49–51], which is the same effect-directionality that can be observed in the PPGR prediction models.

We found that changing the model input slightly in terms of substituting some features for other features ranking in the top of the feature selection models, while still including a similar number of features (models not presented), yielded proper predictions emphasizing the importance of all of the top-ranking features in the feature selection models. Correlating the features included in the combined model demonstrated significant linear associations with PPGRs for all the clinical features, whereas none of the microbiome features did so unless being logarithmically transformed. This emphasizes the need for non-linear modelling of microbiome data.

The model with only bio-clinical features and the model with both bio-clinical features and microbial features had similar predictive performance. The observations can be interpreted as the microbial features are proxies for the bio-clinical features and without any additional measurable effect for the targeted host physiology. Still the observations were expected due to the choice of feature selection method by which ideally all relevant features including the microbiome features are captured even though these may appear less important with smaller effect sizes. The key question of the present study was to investigate the drivers of the inter-individual PPGRs with particular focus on the gut microbiome. Indeed, the finding that an algorithm comprised of abundance of bifidobacteria and the strain MGS.hg0341, annotated to *Clostridiales* order and MGS richness can by themselves predict 14% of the PPGR variance is intriguing.

The main strength of the present study is the transparent reporting of the statistical model including the prior feature selection results. We were able to predict a considerable PPGR variance despite the limited number of samples and features included in the model. By presenting features’ impact and directionality of effects in the model, we here attempted to shed light upon the contributions from the microbiome in a PPGR prediction model. This enables identification of the most important features and can assist in outlining novel hypotheses for further research. Another strength of the present study is the determination and application of QMP in our analyses allowing for characterization of the gut microbiome in terms of absolute abundance. From the microbial loads, information on the extent or directionality of differences in abundance of taxa or functional potential is possible and we are free of relative abundances, which have been shown to underpin both microbiota variation between individuals and covariation with host phenotype [22]. An additional strength of the study was the use of a standardized breakfast mimicking a real life setting in a controlled environment. This allows for a generalizability that goes beyond the standard oral glucose tolerance test.

The main limitation of the present study is the limited number of samples, which increases the risk of overfitting. To minimize overfitting, we limited the number of features in the model and performed fivefold cross-validation. Despite these efforts, the models still overfitted to some extent, as indicated by the decrease in performance in the test set compared to the training set. Ideally, with an adequate number of study participants, we would have divided our samples into training, test and validation sets, respectively. Also, applying this approach in a larger dataset would offer the opportunity to assess the optimal number of features in the models by increased model performance in the test set when adding a feature to the model of interest instead of arbitrarily choosing a number based on simulation studies in the literature. Despite overfitted models, the performance of the models (trained on non-permutated data) was much higher compared with the model trained on permuted data, thus indicating robustness in the presented models. While reducing the number of features in the model decreases the risk of overfitting, it also decreases the accuracy in predicting PPGRs since a large number of microbial features with a small effect is contributing to the variation in PPGRs. In this regard, the enormous interindividual differences in the gut microbiota composition and function demand large cohorts in order to grasp the effects of the wide scale of microbiome features on PPGRs across individuals. Given that each individual has a unique gut microbiota, it may be more feasible and clinically relevant to predict PPGRs within an individual. Unfortunately, in the present study, we cannot differentiate inter- and intra-individual differences, since the cross-sectional design of the study did not allow inclusion of information on PPGRs from the same individual at different time points. For the same reasons, we cannot deduce whether the features in the models are predictors (indicating a potential causal effect) or just associated variables. Bias may also have been introduced during our hypothesis-driven feature selection approach since it relies on *a priori* knowledge. Nevertheless, including the explorative approach has settled this issue to some extent. A possible challenge in the explorative feature selection process is that the majority of the features relate to the gut microbiome, which may have biased the analysis towards microbiome-related features. These features may pose a higher risk of running model overfitting due to the high number and small effect sizes. However, the results from the feature selection of the permuted PPGRs yielded almost exclusively microbiome features, whereas for the explorative feature selection process there was a higher representation of clinical features. This may suggest robustness of the feature selection method against bias related to the high number of microbiome-related features.

The analytical methodology applied in the present study has additional limitations since the plasma glucose excursions based on five time points are summarized as the AUC with the resulting omission of the dynamic information. Another insightful phenotyping may appear from clustering the responses according to glucose curve pattern. Recent studies using latent class trajectory analysis have shown that the trajectory of the glucose curve during an oral glucose tolerance test is predictive of incident diabetes likely reflecting differences in underlying physiology [52]. Moreover, clustering the responses into categories may contribute to a more robust model due to change of random forest model from regression to classification. Yet, identifying biological meaningful curves and ideally compare these to an endpoint is not be possible given the limited sample size and the cross-sectional design of the present study. Even though the PPGR calculated as a single continuous variable cannot reflect the course of the response curve, it has been associated with and manifested as a marker of type 2 diabetes and cardiovascular disease [2, 53] and used as the response variable in other similar studies [3].

Yet, another limitation of the analyses is the requirement of complete cases, which we handled by median imputation of missing data. Imputation increases uncertainty of the output; however, we believe the data is missing at random and median imputation has been reported to yield acceptable accuracy [54]. In addition, confounders such as medication, exercise and genetics known to have an impact on glucose regulation and the gut microbiome were not controlled for and may have distorted the results. Finally, generalizability of the results is limited to a population similar to the study population and the findings thus require reproduction and validation from independent but comparable study samples.

## Supporting information

S1 Fig Interindividual differences in the postprandial plasma glucose responses. The coloured line represents each individual trajectory of measured plasma glucose concentration (n = 106).
(TIF)

S2 Fig Features included in hypothesis-driven approach and ranked by importance in random forest models predicting postprandial plasma glucose responses. Number of times each feature appeared in 200 Boruta rounds, each performing a top-down search for important features by comparing original hypothesis features’ importance with importance achievable at random using random forest models predicting postprandial plasma glucose responses (n = 75). Features that did not appear are not shown.
(TIF)

S3 Fig Association between predicted and measured postprandial plasma glucose responses based on the hypothesis-based model. The black line represents the fitted regression line and the grey shaded area represents the 95% CI. (A) The association in the training set (n = 75). R = 0.99, 95% CI: 0.98 to 0.99, *p*<0.001. (B) The association in the test set (n = 31). R = 0.42, 95% CI: 0.07 to 0.67, *p* = 0.02.
(TIF)

S4 Fig Features included in the explorative approach ranked by importance in random forest models predicting postprandial plasma glucose responses. Number of times each feature appeared in 200 Boruta rounds, each performing a top-down search for important features by comparing all original features’ importance with importance achievable at random using random forest models predicting postprandial plasma glucose responses (n = 75). Features that did not appear are not shown.
(TIF)

S5 Fig Association between predicted and measured postprandial plasma glucose responses based on the explorative model. The black line represents the fitted regression line and the grey shaded area represents the 95% CI. (A) The association in the training set (n = 75). R = 0.91, 95% CI: 0.86 to 0.94, *p*<0.001. (B) The association in the test set (n = 31). R = 0.66, 95% CI: 0.40 to 0.82, *p*<0.001.
(TIF)

S6 Fig Association between predicted and measured postprandial plasma glucose responses based on the model predicting permuted postprandial glucose responses. The black line represents the fitted regression line and the grey shaded area represents the 95% CI. (A) The association in the training set (n = 75). R = 0.99, 95% CI: 0.98 to 0.99, *p*<0.001. (B) The association in the test set (n = 31). R = -0.13, 95% CI: -0.47 to 0.25, *p* = 0.51.
(TIF)

S7 Fig Pearson correlations of postprandial plasma glucose responses and features included in the combined model. The intensity of the colour represents the direction of the correlation with blue corresponding to a positive association. The stars indicate the strength of the correlation. (A) Untransformed data, where fasting serum concentration of triglycerides, MGS.hg0341 and bifidobacteria abundance, respectively, displays non-normal distributions (n = 106). (B) Triglycerides, MGS.hg0341 and bifidobacteria are logtransformed (n = 106).
(TIF)

S8 Fig Features ranked by importance in random forest models predicting permuted postprandial plasma glucose responses. Number of times each feature appeared in 200 Boruta rounds, each performing a top-down search for important features by comparing original features’ importance with importance achievable at random using random forest models predicting postprandial plasma glucose responses (n = 75). Features that did not appear are not shown.
(TIF)

S9 Fig Association between predicted and measured postprandial plasma glucose responses based on the model including microbiome features solely. The black line represents the fitted regression line and the grey shaded area represents the 95% CI. (A) The association in the training set (n = 75). R = 0.64, 95% CI: 0.48 to 0.76 *p*<0.001. (B) The association in the test set (n = 31). R = 0.37, 95% CI: 0.02 to 0.64 *p* = 0.04.
(TIF)

S10 Fig Association between predicted and measured postprandial plasma glucose responses based on the combined model including glycaemic features. The black line represents the fitted regression line and the grey shaded area represents the 95% CI. (A) The association in the training set (n = 75). R = 0.96, 95% CI: 0.93 to 0.97, *p*<0.001. (B) The association in the test set (n = 31). R = 0.78, 95% CI: 0.59 to 0.89, *p*<0.001.
(TIF)

S11 Fig Comparison of the association strengths between the actual and predicted postprandial glucose responses in the test set based on the five different models and a perfect correlation as a comparison (exact); the combined model including glycaemic variables in the fasting state, the combined model, the bio-clinical features-only model, microbiome-only model and the null model of the permuted glucose responses. The dots represent the model predictions for each individual. Exact: Pearson R = 1. The combined model including glycaemic variables in the fasting state: Pearson R = 0.78, 95% CI: 0.59–0.89 and *p*<0.001. The combined model: Pearson R = 0.69, 95% CI: 0.45–0.84 and *p*<0.001. The bio-clinical features-only model: Pearson R = 0.69, 95% CI: 0.45–0.84 and *p*<0.001. The microbiome-only model: Pearson R = 0.37, 95% CI: 0.02–0.64 and *p* = 0.04. The null model of the permuted glucose responses: Pearson R = -0.13, 95% CI: -0.47.0.25 and *p* = 0.51.
(TIF)

S12 Fig Comparison of the association strengths between the actual and predicted postprandial glucose responses in the training set based on the five different models and a perfect correlation as a comparison (exact); the combined model including glycaemic variables in the fasting state, the combined model, the bio-clinical features-only model, microbiome-only model and the null model of the permuted glucose responses. (A) The lines represent the fitted regression lines and the corresponding shaded area represent the 95% CIs for each model, respectively. Exact: Pearson R = 1. The combined model including glycaemic variables in the fasting state: Pearson R = 0.96, 95% CI: 0.93–0.97 and *p*<0.001. The combined model: Pearson R = 0.90, 95% CI: 0.84–0.93 and *p*<0.001. The bio-clinical features-only model: Pearson R = 0.82, 95% CI: 0.73–0.88 and *p*<0.001. The microbiome-only model: Pearson R = 0.64, 95% CI: 0.48–0.76 and *p*<0.001. The null model of the permuted glucose responses: Pearson R = 0.99, 95% CI: 0.98–0.99 and *p*<0.001. (B) The dots represent the model predictions for each individual. Exact: Pearson R = 1. The combined model including glycaemic variables in the fasting state: Pearson R = 0.96, 95% CI: 0.93–0.97 and *p*<0.001. The combined model: Pearson R = 0.90, 95% CI: 0.84–0.93 and *p*<0.001. The bio-clinical features-only model: Pearson R = 0.82, 95% CI: 0.73–0.88 and *p*<0.001. The microbiome-only model: Pearson R = 0.64, 95% CI: 0.48–0.76 and *p*<0.001. The null model of the permuted glucose responses: Pearson R = 0.99, 95% CI: 0.98–0.99 and *p*<0.001.
(TIF)

S1 Table Features including names and numbers within the categories phenomics, biochemical features, lifestyle data, microbial characteristics, microbial taxa and microbial functional modules included in the hypothesis and explorative approach, respectively. (XLSX)

S2 Table Thirty-six features relevant for postprandial plasma glucose responses based on hypotheses. Shown are the feature’s distributions in the study population as mean (SD) or median (IQR) when not normally distributed. Prevalence is presented as number of count and percentage. The direction of the hypothesized association with postprandial plasma glucose response is indicated by +/- with relevant references for each feature.
(XLSX)

S3 Table Features of the hypothesis-driven approach and ranked by importance in random forest models predicting postprandial plasma glucose responses. Final results of feature selection from 200 Boruta runs each performing a top-down search for relevant features based on hypothesized relevance by comparing original features’ importance with importance achievable at random using random forests predicting postprandial plasma glucose response.
(XLSX)

S4 Table Characteristics of the hypothesis-driven random forest model including default variable importance. Hyperparameter names correspond to parameter names from the H2O package in R. Training and test evaluations as the mean squared error, root mean squared error, mean absolute error, root mean squared logarithmic error, mean of the residuals, correlation between predicted and measured postprandial plasma glucose concentrations including confidence intervals and P values, respectively, are presented.
(XLSX)

S5 Table Features of explorative approach and ranked by importance in random forest models predicting postprandial plasma glucose responses. Final results of feature selection from 200 Boruta runs each performing a top-down search for all features by comparing original features’ importance with importance achievable at random using random forests predicting postprandial glucose response.
(XLSX)

S6 Table Characteristics of the explorative random forest model including default variable importance. Hyperparameter names correspond to parameter names from the H2O package in R. Training and test evaluations as the mean squared error, root mean squared error, mean absolute error, root mean squared logarithmic error, mean of the residuals, correlation between predicted and measured postprandial plasma glucose concentrations including confidence intervals and P values, respectively, are presented.
(XLSX)

S7 Table Characteristics of the combined random forest model including default variable importance. Hyperparameter names correspond to parameter names from the H2O package in R. Training and test evaluations as the mean squared error, root mean squared error, mean absolute error, root mean squared logarithmic error, mean of the residuals, correlation between predicted and measured postprandial plasma glucose concentrations including confidence intervals and P values, respectively, are presented. Feature importances including the corresponding confidence intervals are based on the training set, and the p values, associated with the feature importances, are calculated from a paired t-test of the absolute values of the delta values between the original and the permutation-based cross-validation predictions.
(XLSX)

S8 Table Model characteristics including default variable importance of random forest models used to determine individual feature contributions. The feature’s importance is calculated by removing the feature of interest from the combined model, retrain, evaluate the model by correlating measured and predicted postprandial glucose responses and subsequently subtract the correlation R from the one obtained from the combined model including all of the features. Hyperparameter names in the models correspond to parameter names from the h2o package in R. Training and test evaluations as the mean squared error, root mean squared error, mean absolute error, root mean squared logarithmic error, mean of the residuals, correlation between predicted and measured postprandial plasma glucose concentrations including confidence intervals and P values, respectively, are presented.
(XLSX)

S9 Table Features ranked by importance in random forest models predicting permuted postprandial plasma glucose responses. Final results of feature selection from 200 Boruta runs each performing a top-down search for relevant features by comparing original features’ importance with importance achievable at random using random forests predicting postprandial glucose response.
(XLSX)

S10 Table Prediction model of permuted postprandial plasma glucose responses. Characteristics of the random forest model including default variable importance. Hyperparameter names correspond to parameter names from the H2O package in R. Training and test evaluations as the mean squared error, root mean squared error, mean absolute error, root mean squared logarithmic error, mean of the residuals, correlation between predicted and measured postprandial plasma glucose concentrations including confidence intervals and P values, respectively, are presented.
(XLSX)

S11 Table Model characteristics including default variable importance of a random forest model based on solely microbiome features included in the combined model. Hyperparameter names correspond to parameter names from the H2O package in R. Training and test evaluations as the mean squared error, root mean squared error, mean absolute error, root mean squared logarithmic error, mean of the residuals, correlation between predicted and measured postprandial plasma glucose concentrations including confidence intervals and P values, respectively, are presented.
(XLSX)

S12 Table Model characteristics including default variable importance of a random forest model based on only bio-clinical features included in the combined model. Hyperparameter names correspond to parameter names from the H2O package in R. Training and test evaluations as the mean squared error, root mean squared error, mean absolute error, root mean squared logarithmic error, mean of the residuals, correlation between predicted and measured postprandial plasma glucose concentrations including confidence intervals and P values, respectively are presented.
(XLSX)

S13 Table Model characteristics including variable importance of the combined random forest model including glycaemic features. Hyperparameter names correspond to parameter names from the H2O package in R. Training and test evaluations as the mean squared error, root mean squared error, mean absolute error, root mean squared logarithmic error, mean of the residuals, correlation between predicted and measured postprandial plasma glucose concentrations including confidence intervals and P values, respectively, are presented.
(XLSX)

S1 Appendix Supplementary methods. (DOCX)

S1 File PPR. (ZIP)
