# Supplementary figures and images for: The intestinal microbiome is a co-determinant of the postprandial plasma glucose response
Source: PLoS One. 2020 Sep 18;15(9):e0238648. doi: 10.1371/journal.pone.0238648 (PMC7500969; doi:10.1371/journal.pone.0238648)

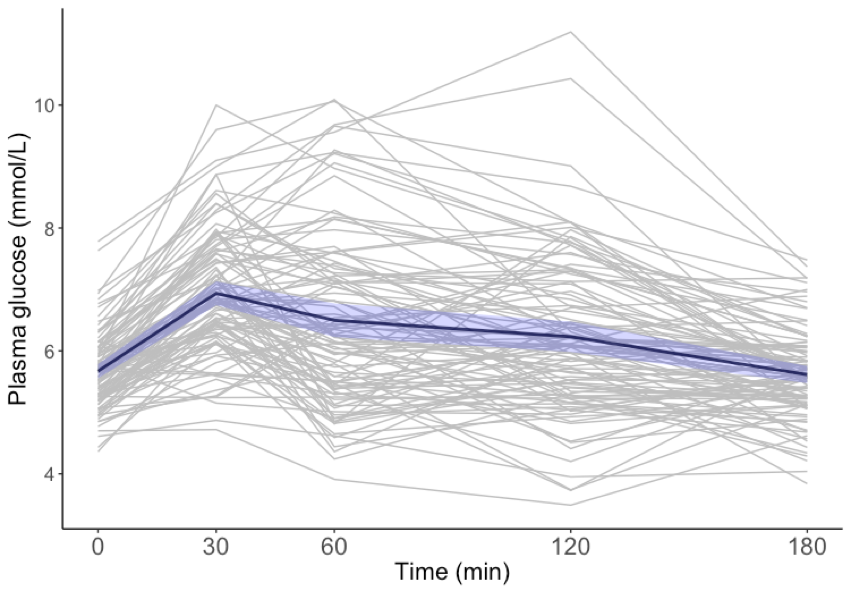

Supplement: S1 Fig — The coloured line represents each individual trajectory of measured plasma glucose concentration (n = 106). (TIF) [file pone.0238648.s001.tif]

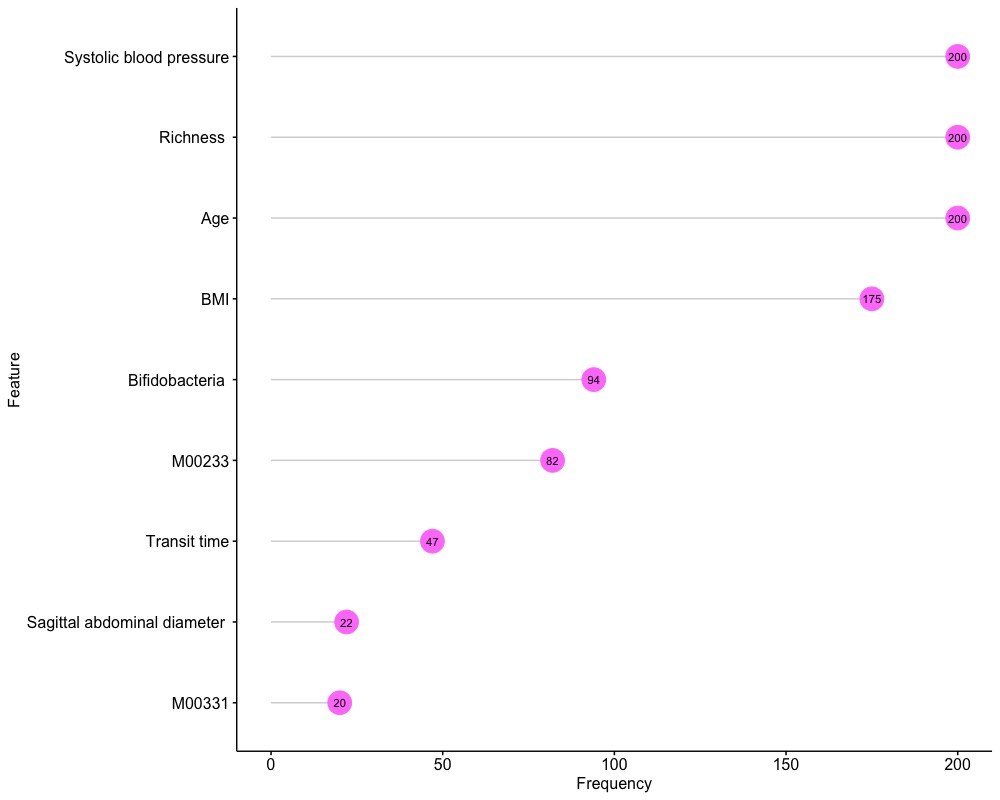

Supplement: S2 Fig — Number of times each feature appeared in 200 Boruta rounds, each performing a top-down search for important features by comparing original hypothesis features’ importance with importance achievable at random using random forest models predicting postprandial plasma glucose responses (n = 75). Features that did not appear are not shown. (TIF) [file pone.0238648.s002.tif]

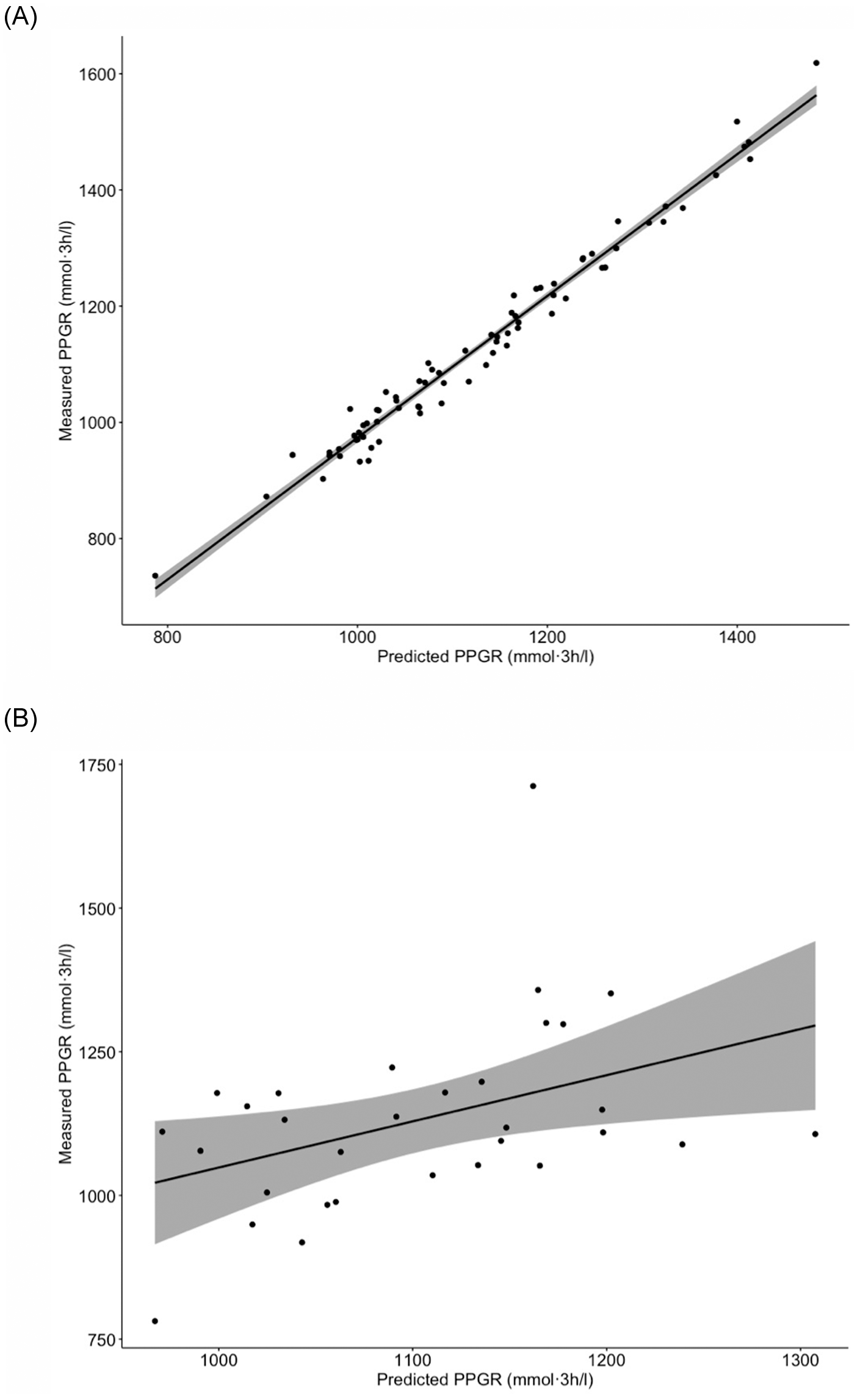

Supplement: S3 Fig — The black line represents the fitted regression line and the grey shaded area represents the 95% CI. (A) The association in the training set (n = 75). R = 0.99, 95% CI: 0.98 to 0.99, p<0.001. (B) The association in the test set (n = 31). R = 0.42, 95% CI: 0.07 to 0.67, p = 0.02. (TIF) [file pone.0238648.s003.tif]

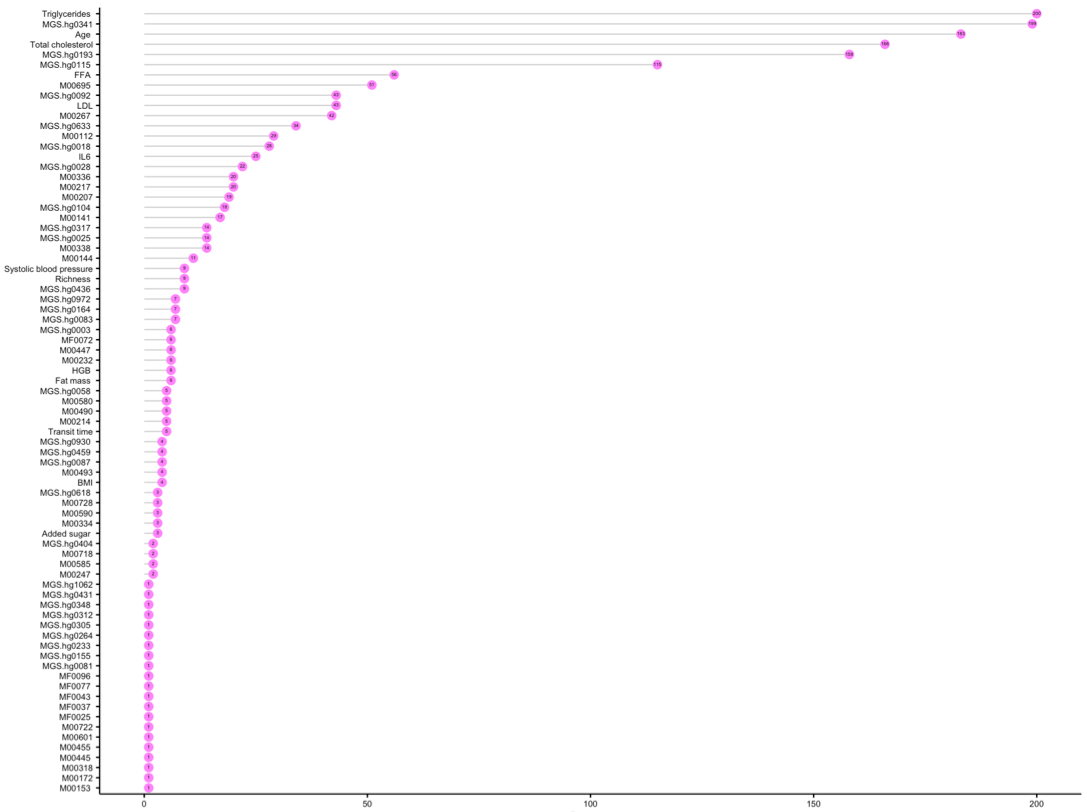

Supplement: S4 Fig — Number of times each feature appeared in 200 Boruta rounds, each performing a top-down search for important features by comparing all original features’ importance with importance achievable at random using random forest models predicting postprandial plasma glucose responses (n = 75). Features that did not appear are not shown. (TIF) [file pone.0238648.s004.tif]

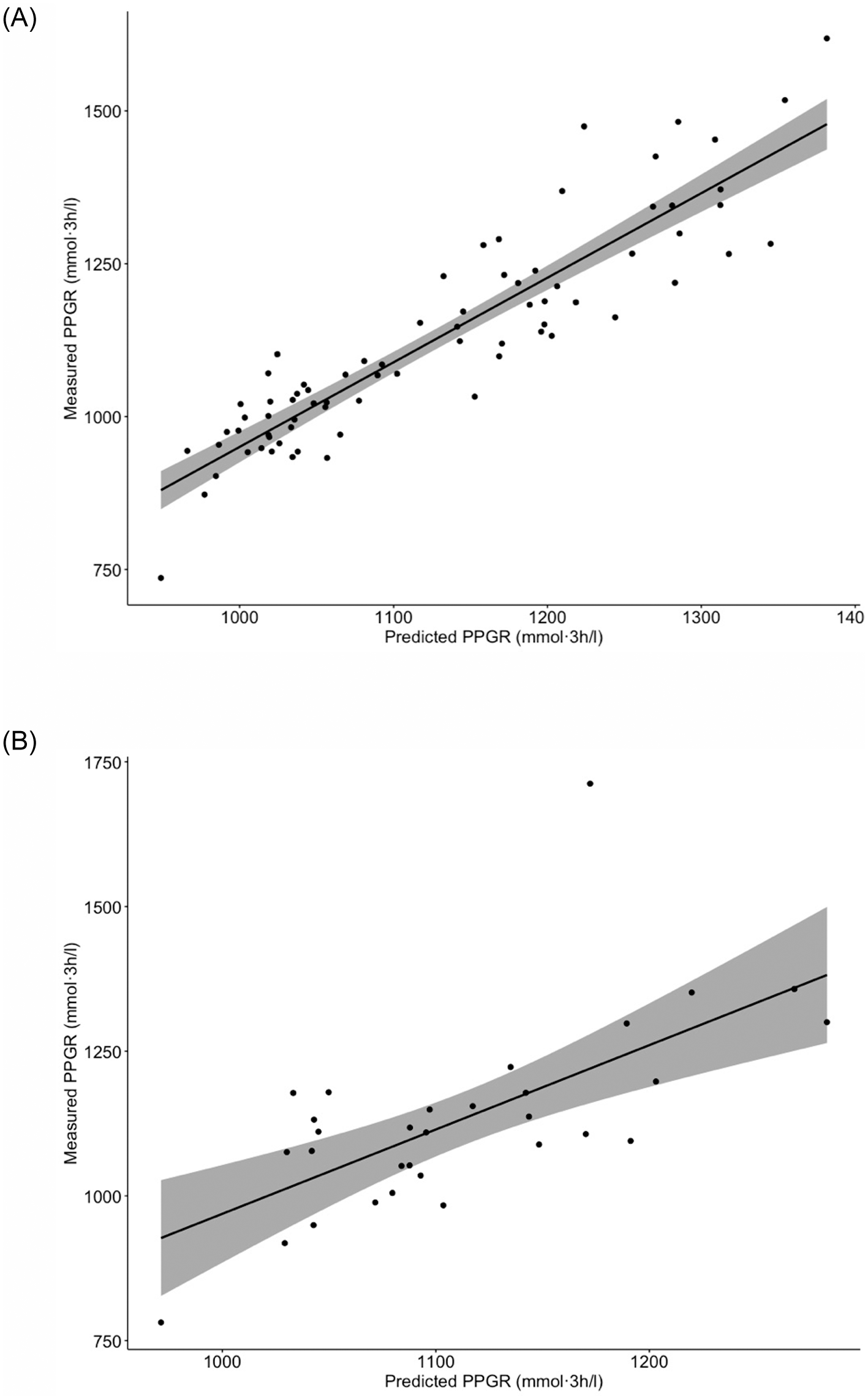

Supplement: S5 Fig — The black line represents the fitted regression line and the grey shaded area represents the 95% CI. (A) The association in the training set (n = 75). R = 0.91, 95% CI: 0.86 to 0.94, p<0.001. (B) The association in the test set (n = 31). R = 0.66, 95% CI: 0.40 to 0.82, p<0.001. (TIF) [file pone.0238648.s005.tif]

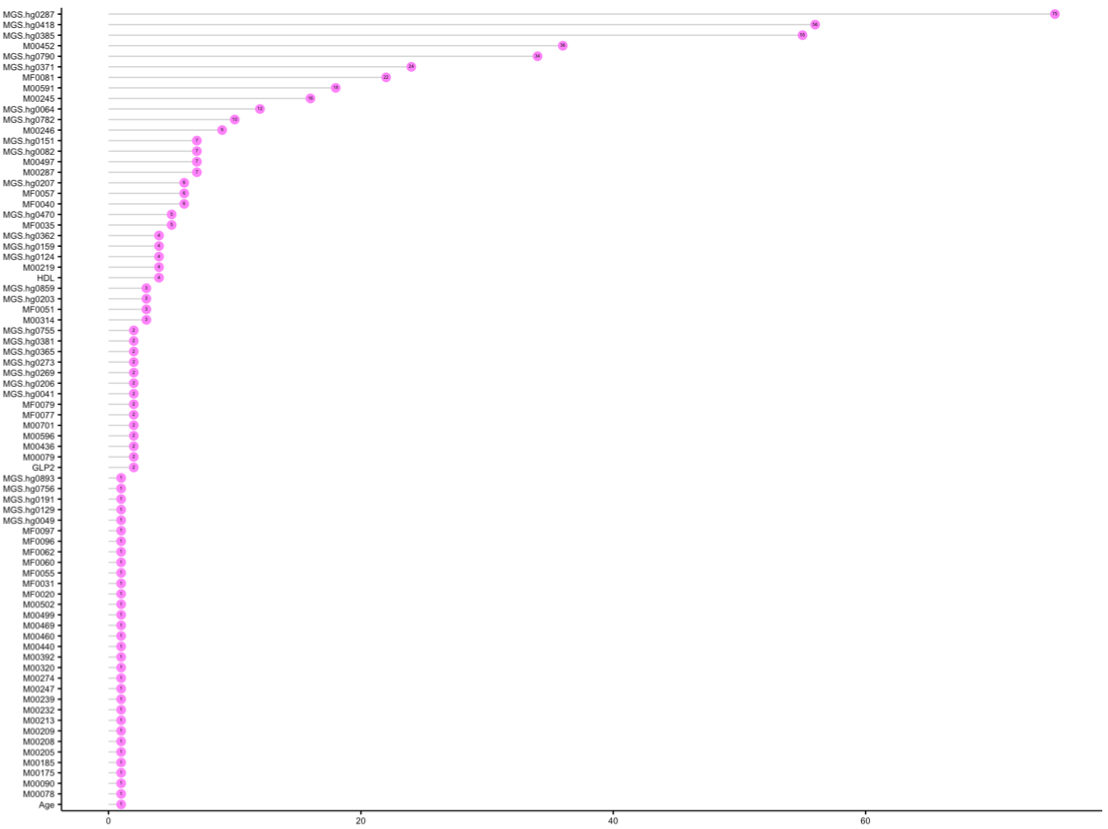

Supplement: S6 Fig — The black line represents the fitted regression line and the grey shaded area represents the 95% CI. (A) The association in the training set (n = 75). R = 0.99, 95% CI: 0.98 to 0.99, p<0.001. (B) The association in the test set (n = 31). R = -0.13, 95% CI: -0.47 to 0.25, p = 0.51. (TIF) [file pone.0238648.s006.tif]

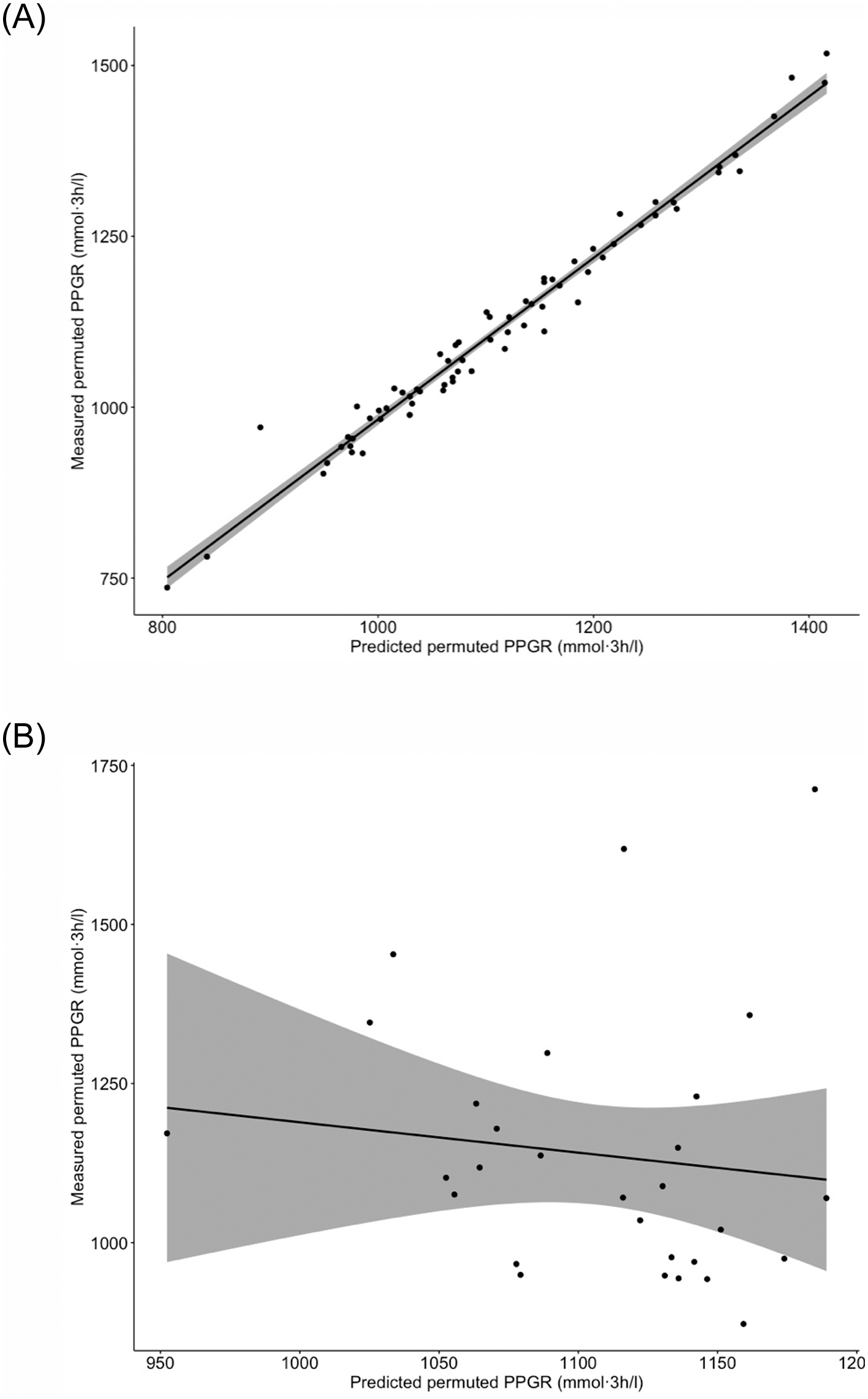

Supplement: S7 Fig — The intensity of the colour represents the direction of the correlation with blue corresponding to a positive association. The stars indicate the strength of the correlation. (A) Untransformed data, where fasting serum concentration of triglycerides, MGS.hg0341 and bifidobacteria abundance, respectively, displays non-normal distributions (n = 106). (B) Triglycerides, MGS.hg0341 and bifidobacteria are logtransformed (n = 106). (TIF) [file pone.0238648.s007.tif]

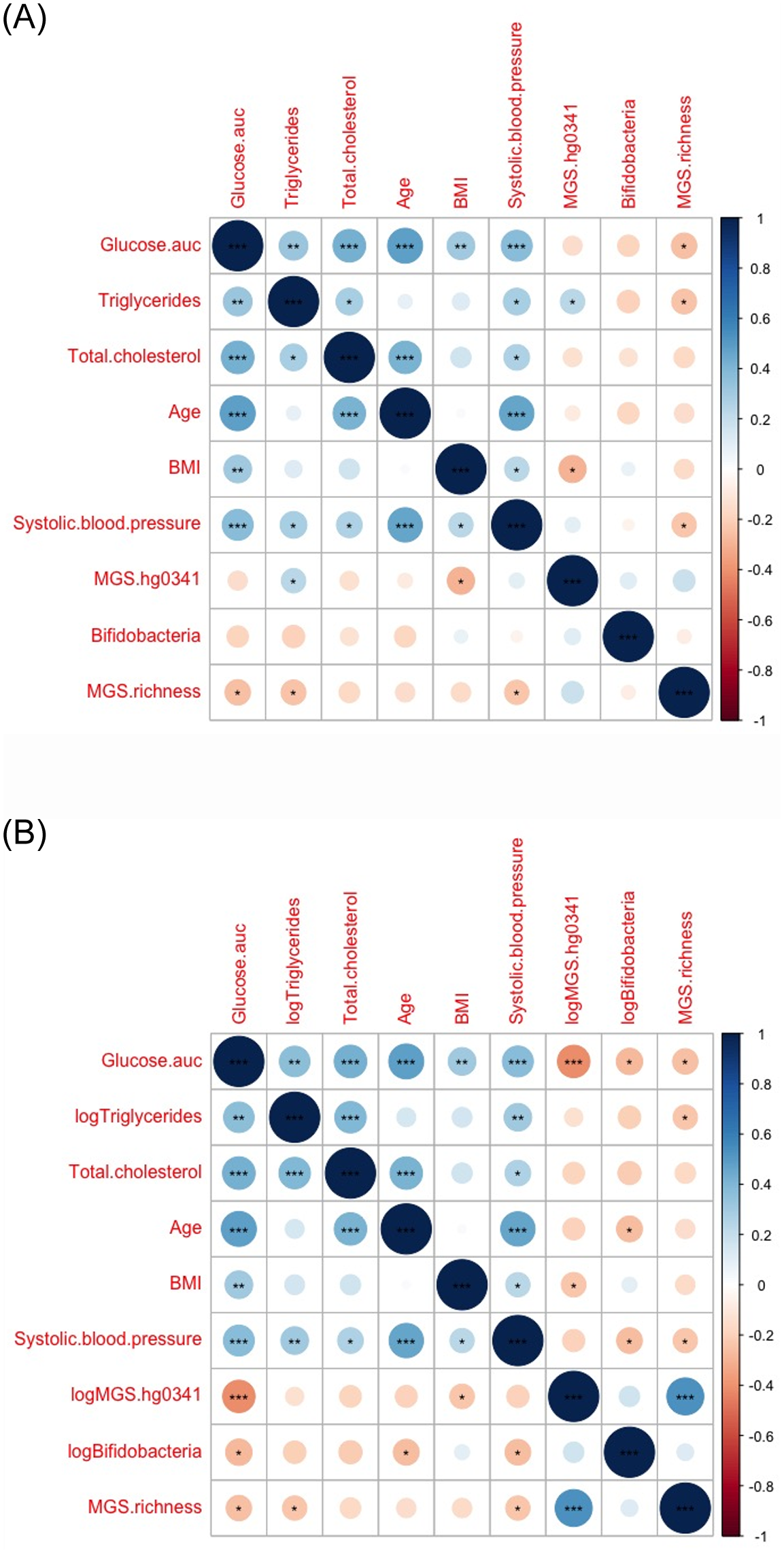

Supplement: S8 Fig — Number of times each feature appeared in 200 Boruta rounds, each performing a top-down search for important features by comparing original features’ importance with importance achievable at random using random forest models predicting postprandial plasma glucose responses (n = 75). Features that did not appear are not shown. (TIF) [file pone.0238648.s008.tif]

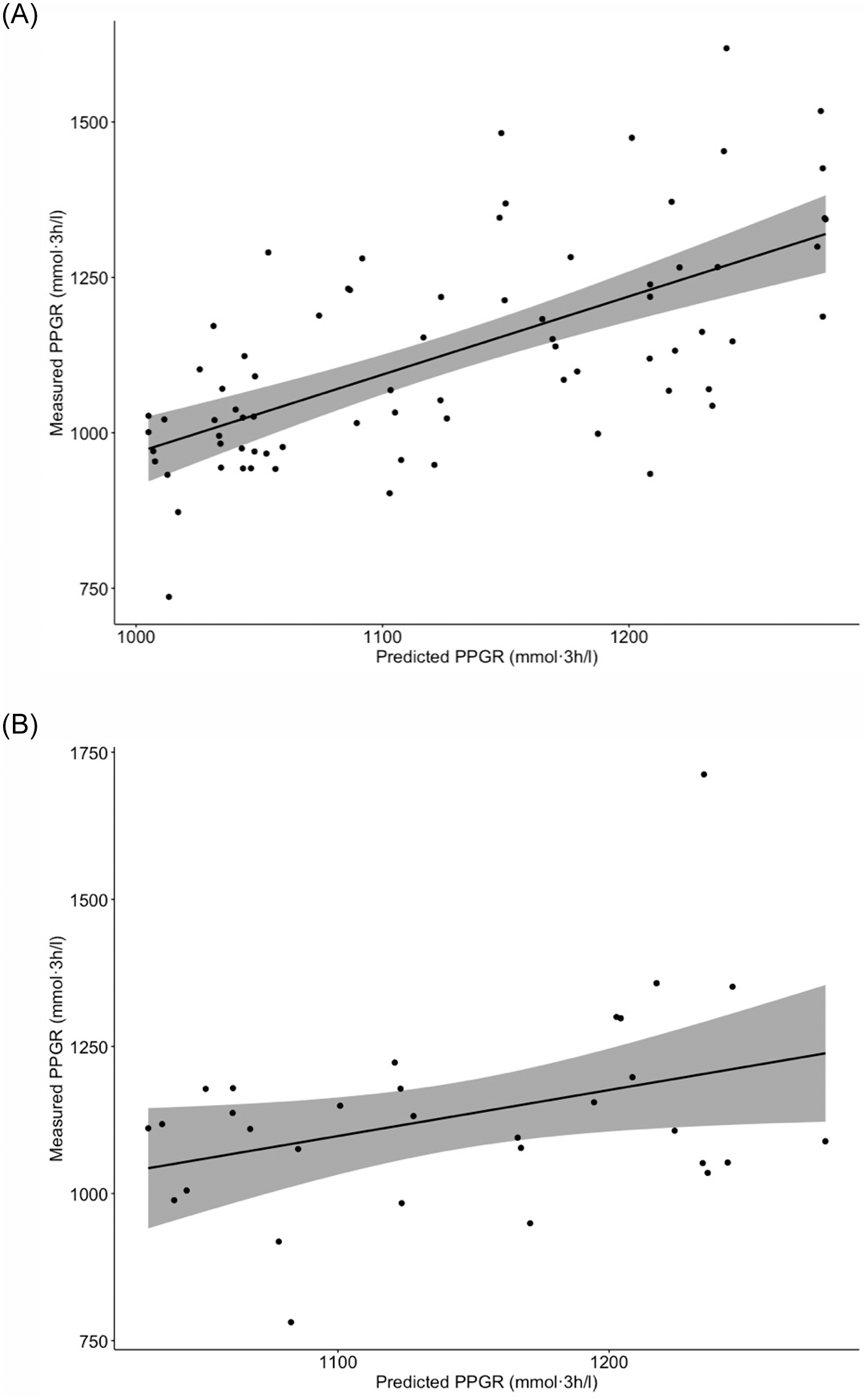

Supplement: S9 Fig — The black line represents the fitted regression line and the grey shaded area represents the 95% CI. (A) The association in the training set (n = 75). R = 0.64, 95% CI: 0.48 to 0.76 p<0.001. (B) The association in the test set (n = 31). R = 0.37, 95% CI: 0.02 to 0.64 p = 0.04. (TIF) [file pone.0238648.s009.tif]

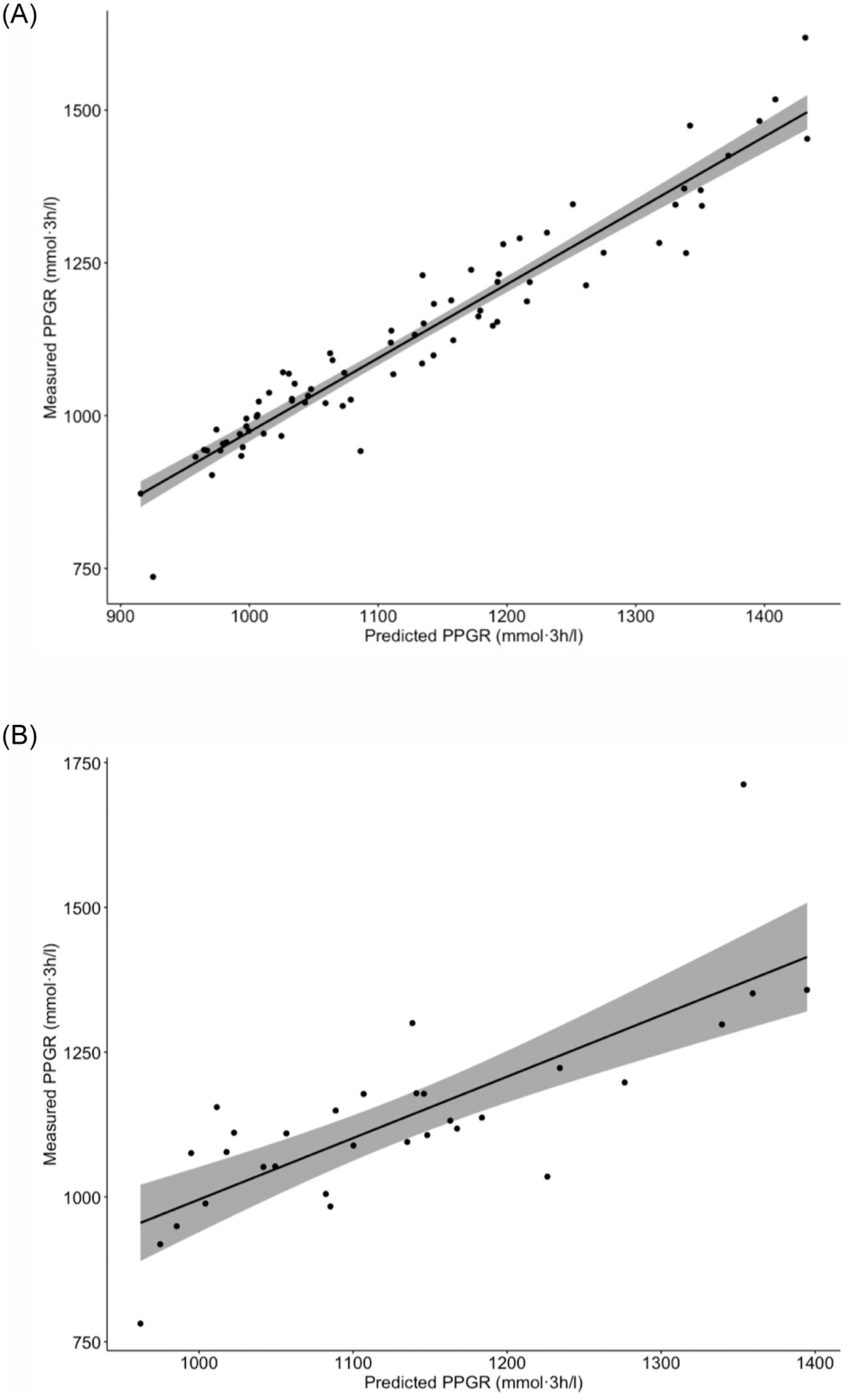

Supplement: S10 Fig — The black line represents the fitted regression line and the grey shaded area represents the 95% CI. (A) The association in the training set (n = 75). R = 0.96, 95% CI: 0.93 to 0.97, p<0.001. (B) The association in the test set (n = 31). R = 0.78, 95% CI: 0.59 to 0.89, p<0.001. (TIF) [file pone.0238648.s010.tif]

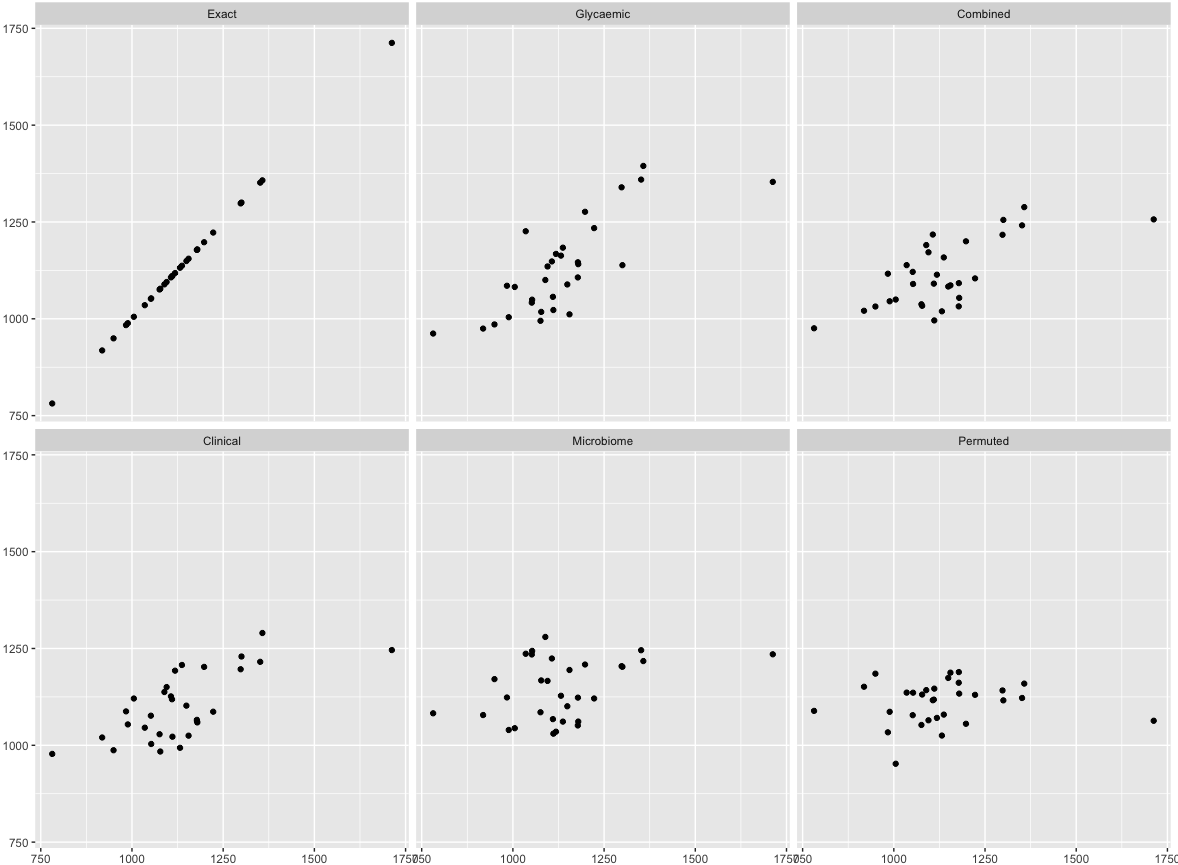

Supplement: S11 Fig — The dots represent the model predictions for each individual. Exact: Pearson R = 1. The combined model including glycaemic variables in the fasting state: Pearson R = 0.78, 95% CI: 0.59–0.89 and p<0.001. The combined model: Pearson R = 0.69, 95% CI: 0.45–0.84 and p<0.001. The bio-clinical features-only model: Pearson R = 0.69, 95% CI: 0.45–0.84 and p<0.001. The microbiome-only model: Pearson R = 0.37, 95% CI: 0.02–0.64 and p = 0.04. The null model of the permuted glucose responses: Pearson R = -0.13, 95% CI: -0.47.0.25 and p = 0.51. (TIF) [file pone.0238648.s011.tif]

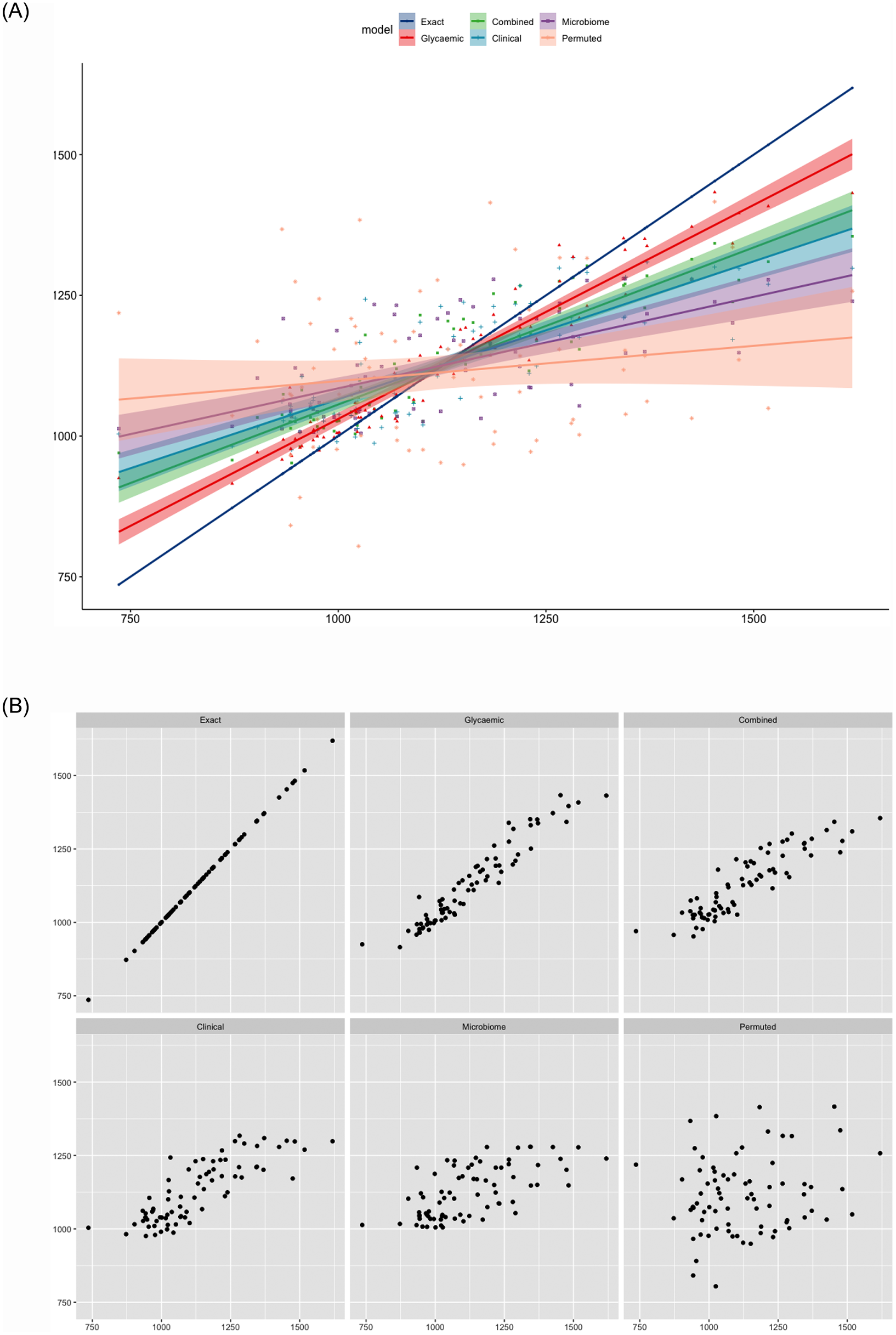

Supplement: S12 Fig — (A) The lines represent the fitted regression lines and the corresponding shaded area represent the 95% CIs for each model, respectively. Exact: Pearson R = 1. The combined model including glycaemic variables in the fasting state: Pearson R = 0.96, 95% CI: 0.93–0.97 and p<0.001. The combined model: Pearson R = 0.90, 95% CI: 0.84–0.93 and p<0.001. The bio-clinical features-only model: Pearson R = 0.82, 95% CI: 0.73–0.88 and p<0.001. The microbiome-only model: Pearson R = 0.64, 95% CI: 0.48–0.76 and p<0.001. The null model of the permuted glucose responses: Pearson R = 0.99, 95% CI: 0.98–0.99 and p<0.001. (B) The dots represent the model predictions for each individual. Exact: Pearson R = 1. The combined model including glycaemic variables in the fasting state: Pearson R = 0.96, 95% CI: 0.93–0.97 and p<0.001. The combined model: Pearson R = 0.90, 95% CI: 0.84–0.93 and p<0.001. The bio-clinical features-only model: Pearson R = 0.82, 95% CI: 0.73–0.88 and p<0.001. The microbiome-only model: Pearson R = 0.64, 95% CI: 0.48–0.76 and p<0.001. The null model of the permuted glucose responses: Pearson R = 0.99, 95% CI: 0.98–0.99 and p<0.001. (TIF) [file pone.0238648.s012.tif]
