# Supplementary material for: The intestinal microbiome is a co-determinant of the postprandial plasma glucose response
Source: PLoS One. 2020 Sep 18;15(9):e0238648. doi: 10.1371/journal.pone.0238648 (PMC7500969; doi:10.1371/journal.pone.0238648)
Supplement: S1 Appendix — (DOCX) [file pone.0238648.s026.docx]

## Quantitative Microbiome Profiling (QMP)

**Cell count.** After the frozen faecal samples were thawed they were divided into 0.08-0.15 g aliquots. The aliquots were frozen (− 80 °C) for further analyses. For cell counting, 0.08-0.15 g aliquots were diluted 2118 times in staining buffer (1 mM EDTA (*Sigma-Aldrich*), 0.01% Tween20 (*Sigma-Aldrich*), pH 7.2 DPBS (*Lonza BioWhittaker*), 1% BSA (*Sigma-Aldrich*)). In order to remove debris from the faecal solutions, samples were filtered using a sterile syringe filter (pore size 5 μm (*pluriSelect*)). Next, 170 μL of the microbial cell suspension obtained was stained with 20 μL DAPI (1mM in H_2_O, *Sigma-Aldrich*).

The flow cytometry analysis of the microbial cells present in the suspension was performed using a BD Fortessa LSRII ﬂow cytometer (*BD Biosciences*). Measurements were performed at pre-set ﬂow rate of 0.5 μL/sec and 100 000 event were recorded per sample. Fluorescence events were monitored using the 440/40 nm, 575/26 nm and 695/40 nm optical detectors. Forward and sideways-scattered lights were also collected. The BD FACSDiva^TM^ Software was used to gate and separate the microbial fluorescence events from the faecal sample background.

A threshold value of 900 was applied on the area of forward scattered (FSC) and a threshold value of 200 was applied on the area of sideways scattered (SSC) channel. Other flow settings are listed as follows.

Supplementary methods table 1 Flow settings to run the flow cytometry analysis

| channel | voltage | … | A | H |
| --- | --- | --- | --- | --- |
| FSC | 475 | √ | √ | √ |
| SSC | 265 | √ | √ | √ |
| 575/26 nm (Pacific blue) | 500 | √ | √ | √ |
| 440/40 nm (Per CP-Cy5-5) | 435 | √ | √ | √ |
| 695/40 nm (PE) | 539 | √ | √ | √ |

Supplementary methods table 2 Flow settings to run the flow cytometry analysis

| flow rate | 0.5 μL/sec |
| --- | --- |
| sample volume | 50 μL |
| mixing volume | 100 μL |
| mixing speed | 180 μL/sec |
| number of mixes | 2 |
| washing volume | 800 μL |
| enable BLR | √ |
| BLR period | 10 |

**Gating strategy.** Density plots of blue ﬂuorescence (440/40 nm) *vs.* FSC allowed for distinction between the stained microbial cells and instrument noise or sample background (Supplementary methods fig. A-C). Density plots of red fluorescence (695/40 nm) *vs.* FSC allowed for distinction between the counting beads and other particles in the testing solution, including bacterials, instrument noise or sample background (Supplementary methods fig D, E). The exact same gates and gating strategy was applied for all samples in the form of a ﬁxed template to allow direct comparison between measured samples.


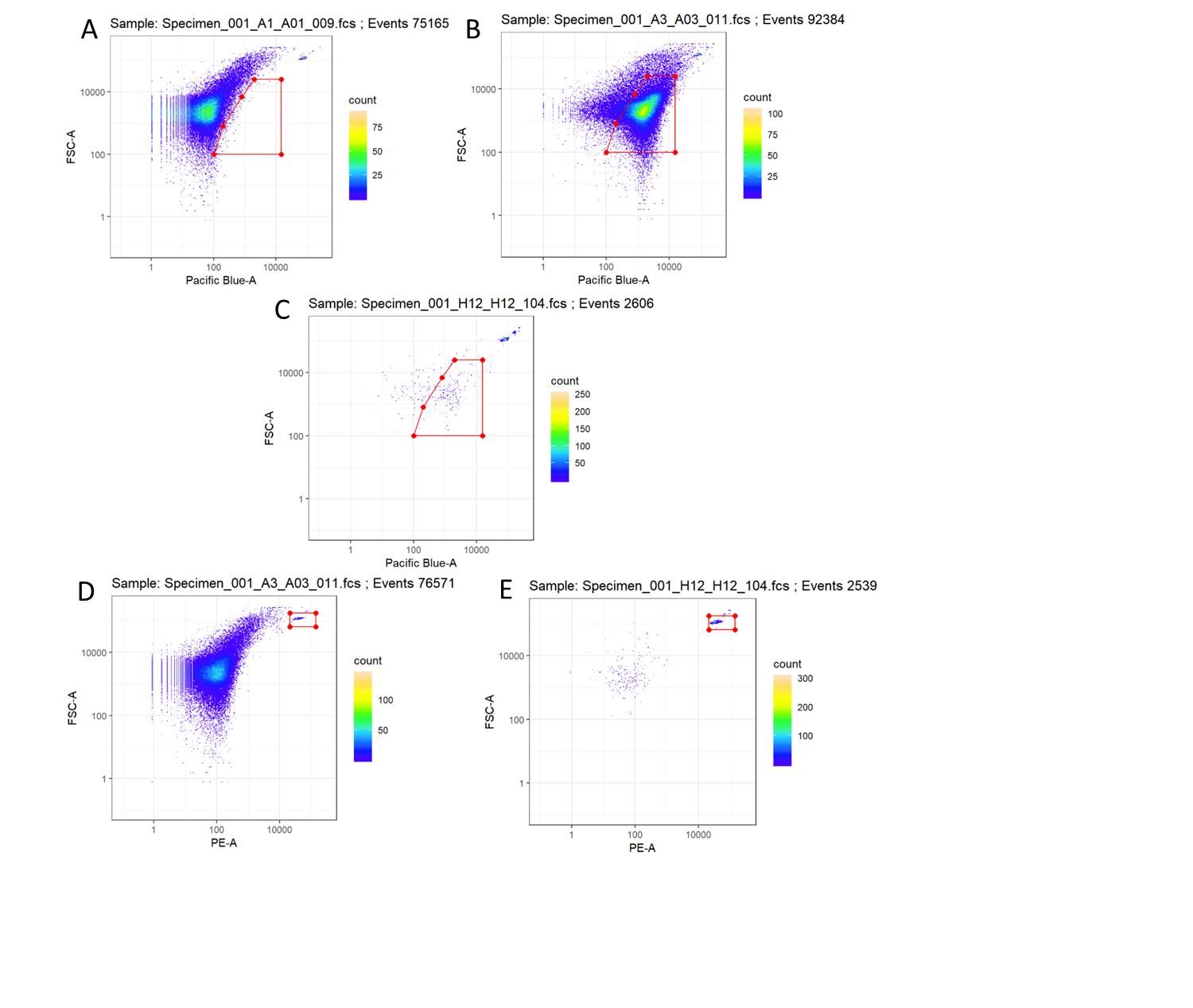

Supplementary methods fig. 1 Density plots

**Gating of the bacterials.** Pacific blue (440/40 nm) vs. FSC allowed for distinction between the stained microbial cells and instrument noise or sample background. A) unstained sample. B) stained sample. C) buffer only sample.

**Gating of the beads.** PE (695/40 nm) *vs.* FSC allowed for distinction between the counting beads and other particles in the testing solution, including bacteria, instrument noise or sample background. D) stained sample. E) buffer only sample.

QMP– rarefaction to equal sampling depth

For quantitative analyses the rarefaction to equal sampling depth is applied(1). Samples are downsized to an even sampling depth, defined as the ratio between sample size and microbial load (average total cell count per gram of frozen faecal material; The sequencing depth of each sample was rarefied to the level necessary to equal the minimum observed sampling depth in the cohort. The R script is available via Raes Lab (<http://www.raeslab.org/software/>).

## Taxonomic and functional annotation of the intestinal microbiome

**Gene catalog and MGS definitions.** As a reference gene catalog, we used the Clinical Microbiomics Human Gut 20M gene catalog (20 041 521 genes), which was created from >5000 publicly available deep-sequenced human gut specimens(2,3,12–18,4–11). For MGS abundance profiling, we used the Clinical Microbiomics HGMGS v.2.3 set of 1273 metagenomic species (MGS), which have highly coherent abundance and base composition in a set of 1776 independent reference human gut samples(19).

### Sequencing data preprocessing

Quality control of raw FASTQ files was performed using KneadData (v. 0.6.1) to remove low-quality bases and reads derived from the host genome as follows: Using Trimmomatic (v. 0.36), the reads were quality trimmed by removing client-specified adapter sequences, leading and trailing bases with a Phred score below 20, and trailing bases in which the Phred score over a window of size 4 drops below 20. Trimmed reads shorter than 90 bases were discarded. Reads that mapped to the human reference genome GRCh38 (with Bowtie2 v. 0.2.3.2 using default settings) were discarded. Read pairs in which both reads passed filtering were retained; these were classified as high-quality non-host reads.

**Mapping reads to gene catalog.** High-quality non-host reads were mapped to the gene catalog using BWA mem (v. 0.7.16a) with options to increase accuracy (-r 1 -D 0.3). PCR/optical duplicates were removed using samtools (v. 1.6). For each individual read, the read was considered mapped if the following criteria were met: an alignment of ≥ 90 bases, ≥ 95 % identity in this alignment, and a mapping quality (MAPQ) ≥ 20. However, if a read failed to align to the gene sequence with > 10 bases at either end, it was considered unmapped. Reads meeting the alignment length and identity criteria but not the MAPQ threshold were considered multimapped. Reads failing the alignment length or identity criteria were considered unmapped.

Read *pairs* were classified into one of three possible categories as follows: 1) Read pairs in which both individual reads were unmapped were considered unmapped. 2) Read pairs in which both individual reads were multimapped, or were mapped to genes in different MGSs, or one was multimapped and the other was unmapped, were considered multimapped. 3) Read pairs in which both individual reads mapped to the same gene; or in which one read mapped to a gene and the other was unmapped, multimapped, or mapped to another gene in the same MGS (see below); were considered mapped. A gene counts table was created with the number of mapped read pairs (for each gene), unmapped read pairs, and multimapped read pairs.

**MGS abundance calculation.** For each MGS, the “core” genes were defined as 100 genes optimized for accurate abundance profiling of the MGS. An MGS counts table was created based on the total gene counts for the 100 core genes of each MGS. However, an MGS was considered detected only if read pairs were mapped to at least three of its 100 core genes; counts for MGSs that did not satisfy this criterion were set to zero.

**Functional annotation and profiling**. Emapper software (v. 1.0.3, HMM mode) was used to compare each gene in the gene catalog to the EggNOG (v. 4.5) orthologous groups database (http://eggnogdb.embl.de/), resulting in annotations for 65% of genes. These genes were then mapped from EggNOG to the Kyoto Encyclopedia of Genes and Genomes orthology database (http://www.genome.jp/kegg/kegg1.html) using MOCAT2 lookup tables (<http://mocat.embl.de/>). The gut metabolic modules annotation was performed in R applying Omixer-RPM (<http://www.raeslab.org/software/gmms.html>).

**Comparison of MGS.hg0341 with known genomes.** A reference human-related gut bacterial genome database comprising nucleotide sequences for each genome was constructed using MultiGeneBlast(20). To further compare MGS.hg0341 sequences with those gut bacterial genomes, we performed multigene homology searches using complete gene sequences against the bacterial genome database by using the stand-alone version of MultiGeneBlast (http://multigeneblast.sourceforge.net/) algorithm with default settings. Subsequently, for queried sequence of MGS.hg0341, we extracted information from the top hit (with the highest cumulative BLAST bit score) from an output of multiple BLAST hits using an in-house script.

## Hyperparameters

**Supplementary Methods Table.** Overview of model hyperparameters that were evaluated. All combinations of listed hyperparameters were tested to identify those that led to the best average AUC using 5-fold cross validation on the training set. Parameter names listed correspond to naming in software implementation (H2O in R)(21).

Supplementary methods table 3

|  | **Grid search** |
| --- | --- |
| **Ntrees** | 100,500 by= 100 |
| **Seed** | 300 |
| **mtries** | 2, XX, by=1 |
| **Max_depth** | 5, 20, by=5 |
| **Min_rows** | 1,5, by=2 |
| **Nbins** | 5,20, by=5 |
| **Sample_rate** | .55, .632, .75 |
| **Strategy** | RandomDiscrete |
| **Stopping metric** | MSE |
| **Stopping tolerance** | 0.005 |
| **Stopping rounds** | 10 |
| **Max runtime secs** | 120*60 |
| **Nfolds** | 5 |

XX is the number of features in the model.

## References

1. Vandeputte D, Kathagen G, D’Hoe K, Vieira-Silva S, Valles-Colomer M, Sabino J, et al. Quantitative microbiome profiling links gut community variation to microbial load. Nature. 2017;551(7681):507–11.

2. Zeevi D, Korem T, Zmora N, Israeli D, Rothschild D, Weinberger A, et al. Personalized Nutrition by Prediction of Glycemic Responses. Cell. 2015;163(5):1079–94.

3. Ferretti P, Pasolli E, Tett A, Asnicar F, Gorfer V, Fedi S, et al. Mother-to-Infant Microbial Transmission from Different Body Sites Shapes the Developing Infant Gut Microbiome. Cell Host Microbe. 2018 Jul;24(1):133-145.e5.

4. Mehta RS, Abu-Ali GS, Drew DA, Lloyd-Price J, Subramanian A, Lochhead P, et al. Stability of the human faecal microbiome in a cohort of adult men. Nat Microbiol. 2018 Mar;3(3):347–55.

5. Costea PI, Coelho LP, Sunagawa S, Munch R, Huerta-Cepas J, Forslund K, et al. Subspecies in the global human gut microbiome. Mol Syst Biol. 2017 Dec;13(12):960.

6. Lloyd-Price J, Mahurkar A, Rahnavard G, Crabtree J, Orvis J, Hall AB, et al. Strains, functions and dynamics in the expanded Human Microbiome Project. Nature. 2017 Oct;550(7674):61–6.

7. Lee STM, Kahn SA, Delmont TO, Shaiber A, Esen OC, Hubert NA, et al. Tracking microbial colonization in fecal microbiota transplantation experiments via genome-resolved metagenomics. Microbiome. 2017 May;5(1):50.

8. Asnicar F, Manara S, Zolfo M, Truong DT, Scholz M, Armanini F, et al. Studying Vertical Microbiome Transmission from Mothers to Infants by Strain-Level Metagenomic Profiling. mSystems. 2017;2(1).

9. Chu DM, Ma J, Prince AL, Antony KM, Seferovic MD, Aagaard KM. Maturation of the infant microbiome community structure and function across multiple body sites and in relation to mode of delivery. Nat Med. 2017 Mar;23(3):314–26.

10. Heintz-Buschart A, May P, Laczny CC, Lebrun LA, Bellora C, Krishna A, et al. Integrated multi-omics of the human gut microbiome in a case study of familial type 1 diabetes. Nat Microbiol. 2016 Oct;2:16180.

11. Yassour M, Vatanen T, Siljander H, Hamalainen A-M, Harkonen T, Ryhanen SJ, et al. Natural history of the infant gut microbiome and impact of antibiotic treatment on bacterial strain diversity and stability. Sci Transl Med. 2016 Jun;8(343):343ra81.

12. Vatanen T, Kostic AD, d’Hennezel E, Siljander H, Franzosa EA, Yassour M, et al. Variation in Microbiome LPS Immunogenicity Contributes to Autoimmunity in Humans. Cell. 2016 Jun;165(6):1551.

13. Korpela K, Salonen A, Virta LJ, Kekkonen RA, Forslund K, Bork P, et al. Intestinal microbiome is related to lifetime antibiotic use in Finnish pre-school children. Nat Commun. 2016 Jan;7:10410.

14. Willmann M, El-Hadidi M, Huson DH, Schutz M, Weidenmaier C, Autenrieth IB, et al. Antibiotic Selection Pressure Determination through Sequence-Based Metagenomics. Antimicrob Agents Chemother. 2015 Dec;59(12):7335–45.

15. Raymond F, Ouameur AA, Deraspe M, Iqbal N, Gingras H, Dridi B, et al. The initial state of the human gut microbiome determines its reshaping by antibiotics. ISME J. 2016 Mar;10(3):707–20.

16. Bengtsson-Palme J, Angelin M, Huss M, Kjellqvist S, Kristiansson E, Palmgren H, et al. The Human Gut Microbiome as a Transporter of Antibiotic Resistance Genes between Continents. Antimicrob Agents Chemother. 2015 Oct;59(10):6551–60.

17. Backhed F, Roswall J, Peng Y, Feng Q, Jia H, Kovatcheva-Datchary P, et al. Dynamics and Stabilization of the Human Gut Microbiome during the First Year of Life. Cell Host Microbe. 2015 May;17(5):690–703.

18. Kostic AD, Gevers D, Siljander H, Vatanen T, Hyotylainen T, Hamalainen A-M, et al. The dynamics of the human infant gut microbiome in development and in progression toward type 1 diabetes. Cell Host Microbe. 2015 Feb;17(2):260–73.

19. Nielsen HB, Almeida M, Juncker AS, Rasmussen S, Li J, Sunagawa S, et al. Identification and assembly of genomes and genetic elements in complex metagenomic samples without using reference genomes. Nat Biotechnol. 2014;32(8):822–8.

20. Medema MH, Takano E, Breitling R. Detecting sequence homology at the gene cluster level with MultiGeneBlast. Mol Biol Evol. 2013 May;30(5):1218–23.

21. LeDell E, Gill N, Aiello S, Fu A, Candel A, Click C, et al. h2o: R Interface for “H2O”. R package version 3.24.0.2. https://github.com/h2oai/h2o-3. 2019;
